# Draft genome of the famous ornamental plant *Paeonia suffruticosa*

**DOI:** 10.1002/ece3.5965

**Published:** 2020-05-12

**Authors:** Shuzuo Lv, Shu Cheng, Zhanying Wang, Shiming Li, Xin Jin, Lei Lan, Bing Yang, Kang Yu, Xuemei Ni, Ning Li, Xiaogai Hou, Gang Huang, Jie Wang, Yang Dong, Erqiang Wang, Jiangtao Huang, Gengyun Zhang, Canjun Zhang

**Affiliations:** ^1^ Luoyang Academy of Agricultural and Forestry Sciences Luoyang Henan China; ^2^ BGI‐Shenzhen Shenzhen China; ^3^ BGI‐Luoyang Agricultural innovation center Luoyang Henan China; ^4^ College of Agriculture Henan University of Science and Technology Luoyang Henan China; ^5^ BGI Institute of Applied Agriculture BGI‐Shenzhen Shenzhen China; ^6^ State Key Laboratory of Agricultural Genomics BGI‐Shenzhen Shenzhen China; ^7^ Key Laboratory of Genomics Ministry of Agriculture BGI‐Shenzhen Shenzhen China

**Keywords:** Comparative genomics, draft genome, MADS‐box, *Paeonia suffruticosa*, Tree peony

## Abstract

Tree peony (*Paeonia* Sect*. Moutan*) is a famous ornamental plant, with huge historical, cultural, and economic significance worldwide. In this study, we reported the ~13.79 Gb draft genome of a wide‐grown *Paeonia suffruticosa* cultivar “Luo shen xiao chun,” representing the largest sequenced genome in dicots to date. Phylogenetic analyses based on genome sequences demonstrated that *P. suffruticosa* was placed as sister to Vitales, and they together formed a clade that was sister to Rosids, weakly supporting a relationship of ((Saxifragales and Vitales) and Rosids). The identification and expression analysis of MADS‐box genes based on the genome assembly and de novo transcriptome assembly of *P. suffruticosa* revealed that the function of C class genes was restricted in flower development, which might be responsible for the stamen petalody in tree peony cultivars. Overall, the first sequenced genome in the family Paeoniaceae provides an important resource for the origin, domestication, and evolutionary study as well as cultivar breeding in tree peony.

## Graphical abstract

Tree peony (Paeonia Sect. Moutan) is a famous ornamental plant, with huge historical, cultural, and economic significance worldwide. The presented genome assembly in this study would serve as an important resource for functional genomic and genetic studies on tree peony, which would be useful for the conservation of endangered wild paeonia species and exploring the mechanisms of plant diversification and adaption under domestication in various environments.

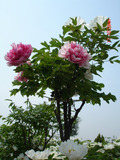

## 1 INTRODUCTION

The genus *Paeonia* is well known for its high ornamental and medical values. It is the only genus in the family Paeoniaceae and consists of 33 species which are assigned to three sections: *Moutan*, *Paeonia,* and *Onaepia* (Christenhusz& Byng, 2016; Ji, Wang, Teixeira da Silva, & Yu, 2012). The tree peonies (*Paenoia* Sect. *Moutan*), native to china, have a long history of cultivation for over 1,600 years (Li, Zhang, & Zhao, 2011). They are perennial deciduous shrubs and were crowned the “king of flowers” for their large and varying forms of flowers, rich and bright colors, symbolizing happiness, wealth, and prosperity in Chinese culture. The seeds of the tree peonies contain rich unsaturated fatty acids such as α‐linolenic acid, oleic acid, and linoleic acid, and are considered to be a novel resource of high‐value edible oil (Li, Wang, et al., 2015a; Li, Yuan, et al., 2015b). *Paeonia suffruticosa* belongs to Section *Moutan*, comprising most of the tree peony cultivars distributed throughout temperate regions in the world (Li et al., 2011). In addition to the ornamental use, the dried root bark of *P. suffruticosa* has been used in Chinese medicine for thousands of years for cardiovascular, extravasated blood, stagnated blood, and female genital diseases (Fu, Yang, Tsai, & Hsieh, 2012).

With long‐term domestication and cultivation as well as natural and artificial selection, there are currently about 2,100 tree peony cultivars worldwide, and China alone has more than 1,000 cultivars (Li et al., 2011). The origin of these cultivars and relationships among them has attracted much attention, but remains unclear due to lack of detailed records of the complicated crossbreeding between wild species and cultivars during the long domestication history (Haw, 2001; Zhou et al., 2014). The numerous different flower colors and shapes of tree peony cultivars represent high genetic diversity and have been used for cultivars’ classification in early studies (Zhou, Zhang, & Zhao, 2007). But based on molecular markers, the genetic groups of cultivars were not necessarily related to flower colors (Guo, Hou, & Zhang, 2009), while different provenances might be the more important factor contributing to the genetic differences (Liu & Lu, 2009; Yuan, Cheng, & Zhou, 2011). The high genetic diversity, along with wide geographic distribution, has made tree peony a fascinating model for studying the mechanisms of diversification and adaptation in plants.

Tree peony has 5 pairs of chromosomes (2n = 10) (Cheng, 2007). The first high‐density genetic map of tree peony was constructed using genotyping by specific‐locus amplified fragment (SLAF) sequencing (Cai, Cheng, Wu, Zhong, & Liu, 2015). It contained 1,189 SLAF markers, spanning 920.699 cM with an average distance of 0.774 cM between adjacent markers. The genetic information of *P. suffruticosa* available to date includes three linkage maps (Cai et al., 2015; Guo et al., 2017; Zhang et al., 2019), 2,415 expressed sequence tags (ESTs) deposited to the NCBI database, and six RNA‐seq datasets. Although analysis of the expressed sequences had contributed a lot to our understanding of the mechanisms of flower bud development (Shu et al., 2009), reblooming (Zhou, Cheng, Wang, Zhong, & He, 2013), prolonging vase life of cut flowers (Zhang et al., 2014), and different color formation in petals (Zhang, Cheng, Ya, Xu, & Han, 2015a) or leaves (Luo, Shi, Niu, & Zhang, 2017), our comprehensive and in‐depth understanding of the genetic basis underlying the numerous flower morphological differences, oil production, and medicinal use is still limited. The availability of a reference genome sequence of *P. suffruticosa* would be helpful for the integration of multi‐omics data across studies to enable more in‐depth research into the biology and genetics of tree peony. Furthermore, a fully annotated genome of *P. suffruticosa* would serve as a foundation for cloning of important horticultural traits‐related genes, identification of new varieties, and conservation of endangered varieties, as well as to promote more efficient breeding of tree peony.

In the present study, we report a de novo assembly and annotation of the *P. suffruticosa* genome, with an estimated genome size of ~13.66–15.76 Gb using PacBio's Single Molecule, Real‐Time Technology (SMRT). Furthermore, we analyzed its phylogenetic relationship with closely related plants based on the genome sequences and reported a comprehensive analyses of the MADS‐box gene family in this tree peony cultivar.

## 2 MATERIAL AND METHODS

### 2.1 Plant materials and sequencing

**Figure 1:**
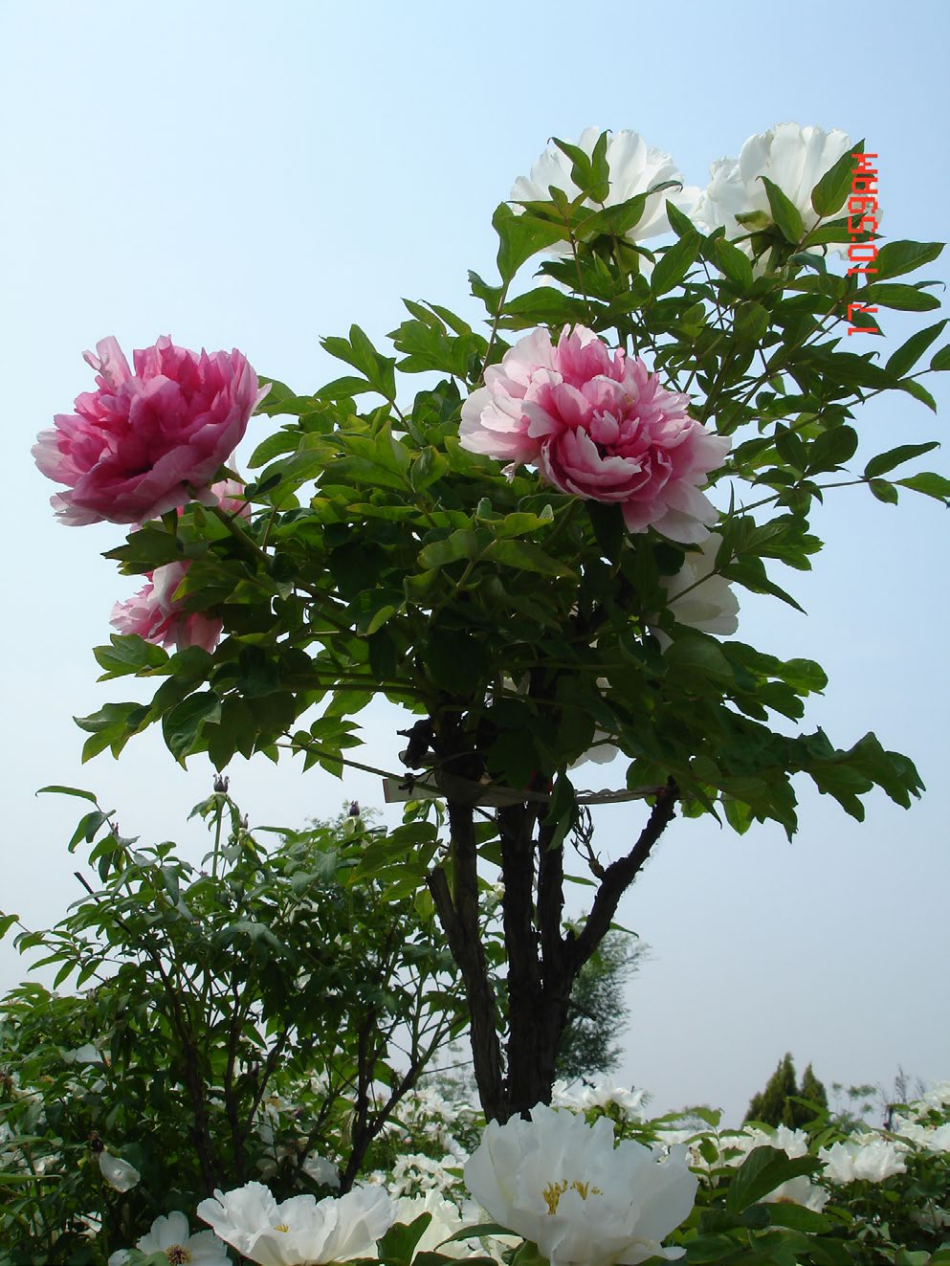
A flowering plant of *P. suffruticosa* “Luo shen xiao chun.”

An individual of *P. suffruticosa* “Luo shen xiao chun” (Figure 1) grown in the peony resource spectrum of Luoyang Academy of Agriculture and Forestry Sciences (N34°39' latitude, E112°27' longitude, Luoyang, China) was selected and the voucher specimen was deposited in the Herbarium of China National GeneBank with a code number “HCNGB_00009295”. The genomic DNA was isolated from the leaves with a standard CTAB extraction method (Murray & Thompson, 1980). A 20kb library was constructed as described previously (Pendleton et al., 2015). Approximately 20 µg of high‐quality genomic DNA was sheared to ~20 kb targeted size and assessed with an Agilent 2,100 Bioanalyzer. Shearing of genomic DNA was followed by damage repair and end repair, blunt‐end adaptor ligation, and size selection with a Blue Pippin system (Sage Science). A total of 114 SMRT cells and 177 SMRT cells were sequenced on PacBio RS II system and PacBio Sequel system, respectively. In total, 96.1 million subreads (894 Gb) were generated with an N50 of 14.5 kb and a mean length of 9.3kb. Furthermore, one paired‐end library was constructed according to the standard protocol provided by BGI (BGI‐Shenzhen) and sequenced on the BGISEQ‐500 platform (Goodwin, McPherson, & McCombie, 2016), with a read length of 100 bp, generating a total of 673 Gb clean data.

Total RNA was extracted from the root, stem, shoot, leaf, flower, and flower bud tissues collected from the same individual using a rapid CTAB‐based method as described previously (Gambino, Perrone, & Gribaudo, 2008). Paired‐end libraries were constructed using standard protocol provided by BGI (BGI‐Shenzhen) and then sequenced on the BGISEQ‐500 platform, with a read length of 100 bp. In total, 45.71 Gb raw data were obtained and after filtering by SOAPnuke (Version 1.5.6) (https://github.com/BGI-flexlab/SOAPnuke), there were 6.85 ~ 8.46 Gb clean data for each sample (Supplementary Table S1).

### 2.2 Estimation of *P. suffruticosa* genome size

A total of 520 Gb high‐quality clean reads obtained from the BGISEQ‐500 platform were subjected to 17, 19, 21, and 23 kmer frequency distribution analyses using Jellyfish (Marcais & Kingsford, 2011). The frequency graph (Supplementary Figure S1) was drawn and the *P. suffruticosa* genome size was calculated using the formula: genome size = kmer_Number/Peak_Depth. For 17 kmer, the total number of kmers was 519,130,610, and 870, and the peak depth was 38. The *P. suffruticosa* genome size was estimated to be 13.66 Gb, and the data used in 17 kmer analysis was about 46.53× coverage of the genome. In 19, 21, and 23 kmer analyses, the genome size was estimated to be 14.35, 14.77, and 15.76 Gb, respectively (Supplementary Table S2).

### 2.3 Genome assembly and completeness assessment

Falcon v1.8.7 (Chin et al., 2016), a diploid‐aware long‐read assembler, was employed to assemble the PacBio subreads in this study. Error correction was first applied to the subreads using parameter “length_cutoff = 13,000, pa_HPCdaligner_option = ‐v ‐B286 ‐t12 ‐w8 ‐M24 ‐e.75 ‐k18 ‐h380 ‐l2800 ‐s1000 ‐T4,” and a total of 288 Gb corrected data were achieved. Then, these corrected reads were used to assemble the genome with parameter “length_cutoff_pr = 9,000, ovlp_HPCdaligner_option = ‐v ‐B180 ‐t12 ‐k18 ‐h180 ‐e.95 ‐l2200 ‐s1000, overlap_filtering_setting = ‐‐max_diff 40 ‐‐max_cov 60 ‐‐min_cov 1.” As a result, 11.8 Gb assembly with the contig N50 length of 76.7 kb was generated (Supplementary Table S4). Since the assembling of highly repetitive genome is sensitive to program parameters in FALCON pipeline, we tuned the parameter values of length_cutoff_pr and overlap_filtering_setting to explore the alternative assemblies (Supplementary Table S3). Finally, we obtained seven different assembly versions (Supplementary Table S4). Among them, the N50 length ranged from 48.4 kb in version 7 to 76.7kb in version 1, and the assembly size ranged from 11.5 Gb in version 2 to 13.8 Gb in version 6. In an overall view, the assembling result showed that no assembly was undoubtedly better than another one. To choose the most suitable genome assembly for functional genomic studies, we further evaluated the completeness of the seven assemblies by comparing them against a set of 1,440 conserved plant genes in BUSCO embryophyta_odb9 dataset using BUSCO v2.0 (Simao, Waterhouse, Ioannidis, Kriventseva, & Zdobnov, 2015) pipeline. The completeness score ranged from 57.5% in version 3 to 61.2% in version 6 (Supplementary Table S5). We observed that the completeness score was not necessarily accordant with the contiguity (contig N50) among the seven assembly versions. Although version 1 has the highest N50 value (76.7 Kb), it captured less genome sequence than other versions and has a relatively smaller completeness score (58.3%). In fact, the assembly version 6 and 7 contained more total sequence than version 1 to 5, which might be due to the set of a smaller value of parameter “length_cutoff_pr = 6,000.” We chose the assembly version 6 for further improvement because it had the highest completeness score and contained the most total sequences. High‐quality BGISEQ‐500 reads were mapped to this assembly with BWA‐MEM (Li, 2013) with default parameters, and high‐quality mapped reads (MAQ >20) were further used to polish the assembly with Pilon (https://github.com/broadinstitute/pilon/wiki) with default parameters. Finally, the obtained assembly was named V_final, which had a total length of 13.79 Gb with an N50 length of 49.94 Kb.

In addition, four publicly available RNA‐seq datasets for tree peonies (NCBI Short Read Archive, accession number: SRX336125, SRX314813, SRX698348, and SRX2439581) were aligned to the final assembly version using BLAT (Kent, 2002) for further validation.

### 2.4 Annotation

Tandem repeats were identified using Tandem Repeats Finder v4.07b (Benson, 1999). For the transposable element annotation, RepeatMasker v3.3.0 (Tarailo‐Graovac & Chen, 2009) and RepeatProteinMasker v3.3.0 (Tarailo‐Graovac & Chen, 2009) were used against Repbase 16.10 (Jurka et al., 2005) to identify known repeats in the *P. suffruticosa* genome. De novo repeat identification was conducted using RepeatModeler v1.0.5 (Price, Jones, & Pevzner, 2005) and LTR_FINDER v1.0.5 (Xu & Wang, 2007) programs, followed by RepeatMasker v3.3.0 to achieve the final results.

Gene models were predicted using a combination of de novo prediction, homology‐based prediction, and transcriptome‐based prediction. For de novo prediction, Augustus (Stanke, Steinkamp, Waack, & Morgenstern, 2004) analysis was conducted on the repeat masked genome, with *Vitis vinfera* as the reference. For homology‐based prediction, protein sequences of *Glycine max* (Schmutz et al., 2010), *Solanum lycopersicum* (Tomato Genome, 2012 *)*, *Vitis vinifera* (Jaillon et al., 2007), *Prunus persica* (International Peach Genome I, 2013), and *Arabidopsis thaliana* (Initiative, 2000) were aligned against *P. suffruticosa* genome using tBLASTn v2.2.26 (E‐value ≦ 1.0e‐05). Gene structure was predicted using GeneWise (Birney, Clamp, & Durbin, 2004). For transcriptome‐based prediction, clean RNA‐seq reads generated in this study were mapped to the assembly using TopHat v2.1.0 (Trapnell, Pachter, & Salzberg, 2009) and assembled into transcripts using Cufflinks (Trapnell et al., 2012), then open reading frames were predicted with hidden Markov model (HMM)‐based training parameters. Results derived from the above methods were integrated by EVM (Haas et al., 2008) to produce a consensus gene set.

The predicted gene models were functionally annotated by aligning their protein sequences against the KEGG (Kanehisa & Goto, 2000), GO (Ashburner et al., 2000), SwissProt (Bairoch & Apweiler, 2000), TrEMBL, and NR protein databases with BLASTP (E‐value ≦ 1.0e‐05). Protein motifs and domains were identified by comparing the sequences against various domain databases, including PFAM, PRINTS, PANTHER, ProDom, PROSITE, and SMART using InterProScan v5.21–60.0 (Quevillon et al., 2005). For ncRNA annotation, tRNA genes were identified by tRNAscan‐SE V1.23 (Lowe & Eddy, 1997). rRNA genes were identified by aligning the rRNA sequences from closely related species (*V. vinifera* and *K. fedtschenkoi*) against the assembly using BLASTN (E‐value ≦ 1.0e‐05). miRNAs and snRNAs were predicted by using INFERNAL (Nawrocki, Kolbe, & Eddy, 2009) software against the Rfam database (Griffiths‐Jones et al., 2005).

### 2.5 Gene family and phylogenetic analysis

For gene family analysis, OrthoMCL (Li, Stoeckert, & Roos, 2003) was used to construct orthologous gene families on all the protein‐coding genes of *P. suffruticosa* and 7 sequenced plant species (*O. sativa* (Ouyang et al., 2007), *S. lycopersicum*, *C. roseus* (Kellner et al., 2015), *G. max*, *P. persica*, and *V. vinifera, Kalanchoë fedtschenkoi* (Yang et al., 2017)). Before OrthoMCL, BLASTP was performed to find similar matches from different species with an E‐value cutoff of 1.0e‐05. The number of gene families in each species was calculated based on the composition of the OrthoMCL clusters. Genes that were single copy in an OrthoMCL cluster for all species analyzed were selected to construct phylogenetic trees using two methods. For the concatenation‐based method, the protein sequences were aligned using PRANK software version 170,427 (http://wasabiapp.org/software/prank/) and was then trimmed using Phyutility version 2.2.6 (Smith & Dunn, 2008) to remove poorly aligned regions with more than 30% missing data. The alignments were concatenated into one supermatrix file, which was used to reconstruct maximum‐likelihood (ML) phylogenetic tree using IQ‐TREE version 1.5.5 (Nguyen, Schmidt, Haeseler, & Minh, 2015) with automatic model selection and 1,000 bootstrap replicates. For coalescence‐based method, individual ML gene trees were reconstructed from the CDS alignments using RAxML version 8.2.11 (Stamatakis, 2014) with GTRGAMMA model and 500 bootstrap replicates. The gene trees were used to reconstruct a species tree using ASTRAL v5.5.9 (Mirarab et al., 2014), with 1,000 bootstraps.

Species divergence times were estimated using MCMCTREE in PAML version 4.9 (Yang, 2007), based on the coalescent phylogenetic tree reconstructed from 511 single copy genes using ASTRAL. The ML estimates of branch lengths were obtained using CODEML programs in PAML under the JONES + gamma substitution models with the gamma priors set at 0.5. Two priors, the overall substitution rate (rgene gamma) and rate‐drift parameter (sigma2 gamma), were set at G (1, 4.3) and G (1, 4.5). The correlated rates were used to specify the prior of rates among internal nodes (clock = 3 in MCMCTREE). The parameters of the birth‐death process for tree generation with species sampling were fixed at BDparas = 1 1 0. A loose maximum bound for the root was set at <10.0 (=1,000 Ma). Markov chain Monte Carlo (MCMC) approximation with a burn‐in period of 5,000,000 cycles was obtained, and every 5,000 cycles was taken to create a total of 10,000 samples. To diagnose possible failure of the Markov chains to converge to their stationary distribution, two replicate MCMC runs were performed with two different random seeds for each analysis. The stationarity of the chains and convergence of two runs were monitored by Tracer version 1.7 ( Rambaut, Drummond, Xie, Baele, & Suchard, 2018) (https://github.com/beast-dev/tracer/releases/tag/v1.7.1). Divergence time estimates in TimeTree (Hedges, Marin, Suleski, Paymer, & Kumar, 2015) database were used for selecting the calibration priors. The lower and upper calibration values were chosen as 77–91, 82–116, and 110–124 for the most recent common ancestor (MRCA) of *C. roseus* and *S. lycopersicum*, *P. persica* and *G. max*, and eudicots, respectively. CAFÉ (De Bie, Cristianini, Demuth, & Hahn, 2006) was used to predict the expansion and contraction of gene family numbers based on the phylogenetic tree and gene family statistics.

### 2.6 Identification of MADS‐box genes from *P. suffruticosa* genome assembly and de novo transcriptome assembly

We identified putative MADS‐box genes in *P. suffruticosa* genome using two methods. First, the 107 *Arabidopsis* MADS‐box Protein sequences were used as query for BLASTP searches against the predicted *P. suffruticosa* protein sequences with an E‐value cutoff of 1.0e‐05. Then, HMMER (Finn, Clements, & Eddy, 2011) searches were performed in *P. suffruticosa* protein sequences using the hidden Markov model (HMM) profiles of MADS‐box domain (PF00319) from the Pfam database (http://pfam.janelia.org). All putative MADS‐box protein sequences obtained by the two methods were manually inspected for removing redundant gene sequences and confirming the existence of MADS‐box domain according to their InterPro annotation. Meanwhile, if a sequence contained K domain in InterPro analysis, we classified it as type II MADS‐box gene, otherwise as type I gene.

The transcriptome data produced in this study were used to search for potentially missing MADS‐box genes. The RNA‐seq reads of the six tissues were filtered using Trimmomatic v 0.38 (Bolger, Lohse, & Usadel, 2014) with parameters “HEADCROP: 15 LEADING: 20 TRAILING: 20 SLIDINGWINDOW: 5:20 MINLEN: 50 AVGQUAL: 20.” Then, the clean reads were assembled using Trinity v2.4.09 (Grabherr et al., 2011) with a minimum contig length setting to 150 bp for each sample. We identified putative coding sequences (CDSs) within each transcript with TransDecoder (https://transdecoder.github.io/) using default parameter settings. We merged the CDSs of all samples and removed redundant sequences using CD‐HIT‐EST v4.6 (Li, 2006) with parameters “‐c 0.8 ‐r 0.” The putative MADS‐box genes were identified from these CDSs using the same methods described above.

To further classify these genes into subfamilies, two individual phylogenetic trees for type I and type II genes were constructed using MADS‐box protein sequences from *P. suffruticosa* and *Arabidopsis*. Multiple sequence alignment was performed using the Clustal X program (Larkin et al., 2007), and phylogenetic trees were then constructed using MEGA5 software (Tamura et al., 2011) with the neighbor‐joining (NJ) method. Bootstrap values were calculated with 1,000 replicates to evaluate the support of the nodes.

### 2.7 Expression analysis of *P. suffruticosa* MADS‐box genes based on genome assembly and de novo transcriptome assembly

The expression profiles of the identified MADS‐box genes in different tissues of *P. suffruticosa* were analyzed using the transcriptome data generated in this study. Because the two gene sets obtained from the genome assembly and from de novo transcriptome assembly were largely different, we calculated the gene expression level for both separately. The filtered clean reads were mapped to the two gene sets using BOWTIE2 v2.2 (Langmead & Salzberg, 2012). The gene expression level was first quantified using RSEM program (Li & Dewey, 2011) and then was normalized by calculating the FPKM value for comparison between different samples.

## 3 RESULTS

### 3.1 Genome assembly and completeness assessment

Based on k‐mer analyses, the *P. suffruticosa* genome was estimated to be ~13.66–15.76 Gb in size (Supplementary Figure S1). It is the largest genome in the sequenced dicots to date and presents a big challenge for genome sequencing and assembly. To assemble the *P. suffruticosa* genome, a total of 894 Gb third‐generation long reads were generated using PacBio RS II system and PacBio Sequel system, representing ~67× coverage of the genome. Although PacBio reads have a relatively high error rate of ∼15%, de novo assembly using these data was proved to be accurate enough with a deep coverage, typically >50× (Berlin et al., 2015). Falcon pipeline (Chin et al., 2016) was used to assemble the genome with significant parameter tuning, and seven different assembly versions were obtained. Completeness assessment of all the assemblies was performed using BUSCO (Simao et al., 2015) to choose the best assembly, followed by polishing with high‐quality short reads. The final assembly version spanned 13.79 Gb in 499, 810 contigs (N50 = 49.94 kb) (Table 1). Completeness assessment showed that 66.1% of the expected 1,440 plant conserved genes were detected as complete (Supplementary Table S5), which was comparable to that of recently sequenced mega‐genomes (>10 Gb), such as 53% of sugar pine (Stevens et al., 2016) and 74% of *Ginkgo biloba* genome (Guan et al., 2016). Additionally, RNA sequence reads generated from six different tissues were mapped to our genome assembly by TopHat v2.1.0 (Trapnell et al., 2009) and the average mapping ratio was 73.2% (Supplementary Table S6). We also mapped four publicly available RNA‐seq datasets to this assembly and found that the mapping ratio was lower (0.04% ~ 68.41%, Supplementary Table S6), indicating high genetic diversity among different *P. suffruticosa* cultivars.

**Table 1:** Statistics of the final genome assembly

Category		Number	N50 (bp)	Size (bp)	Percentage of the assembly (%)
Contigs		499,810	49,937	13,793,297,086	100.00
Repetitive sequence				11,054,226,421	80.24
Transposable elements	LTR	—		6,874,219,419	49.90
	DNA			1,861,990,994	13.52
	LINE			1,931,149,253	14.02
	SINE			157,802,455	1.15
	Unknown			2,058,919,316	14.95
Annotated genes	35,687			6747/210/1192^a^	

a Average mRNA length, exon length, and intron length, respectively.

### 3.2 Repeat analysis and gene prediction

In total, 11.05 Gb of repeat elements were identified, accounting for 80.24% of the 13.79‐Gb genome assembly. Like other plant genomes, the long terminal repeat retrotransposons were the most abundant class of repetitive elements (49.9% of the assembled sequences), of which two superfamilies, Gypsy and Copia, account for 38.91% and 5.12% of the genome assembly, respectively (Supplementary Table S7). DNA class repeat elements represented 13.52% of the genome. Using RepeatMasker, we found that the sequence divergence rates of LTR‐RTs were about 14 ~ 18% higher than other classes of transposon elements (Supplementary Figure S2), indicating that LTR‐RTs played an import role in the genome evolution of tree peony.

By integrating gene prediction results from ab initio, homology‐based, and transcripts‐based approaches, we predicted a nonredundant set of 35,687 gene models with an average gene length of 6,747 bp and an average coding sequence of 1,188 bp (Table 1; Supplementary Table S8). Of the 35,687 predicted genes, 51.37% were supported by either the identification of homologues in other species or RNA‐seq data. The average lengths of gene, CDS, introns, and exons in *P. suffruticosa* were compared with selected six eudicots and were found to be similar to those reported for *V. vinifera* genome, indicating a relative close relation between them (Supplementary Table S9; Supplementary Figure S3). Functions were assigned to 34,854 (97.67%) genes, of which 32,258 (90.39%) had homology to proteins in SwissProt (Bairoch & Apweiler, 2000) and 31,885 (89.35%) had known protein domains in InterPro (Quevillon et al., 2005) (Supplementary Table S10). In addition to protein‐coding genes, we predicted 1,215 tRNA, 1,510 rRNA, 960 microRNA (miRNA), and 1,055 small nuclear RNA (snRNA) genes in our assembly (Supplementary Table S11).

### 3.3 Gene families and phylogenic reconstruction

Using OrthoMCL (Li et al., 2003), the predicted 35,687 protein‐coding genes in *P. suffruticosa* were assigned into 10,882 gene families consisting of 22,279 genes, while 13,408 genes were not organized into groups which might be mis‐annotated or derived from lineage‐specific expansion. Among these gene families, 1,794 were unique in *P. suffruticosa* compared with other seven plant species (Supplementary Table S12).

To infer phylogenetic relationships, a total of 511 single copy orthologs corresponding to the eight species were extracted from the clusters and were used to reconstruct phylogenetic trees with coalescence‐based and concatenation‐based method, respectively (Supplementary Figure S4a,b). The resulting species trees demonstrated a conflict on the placement of *K. fedtschenkoi*. In the species tree reconstructed from concatenated protein sequences, *K. fedtschenkoi* was placed as sister to the eudicots. In the coalescent species tree, *K. fedtschenkoi* was placed as sister to a clade of Vitales + Rosids, which was consistent with the APG IV tree (Chase et al., 2016). However, in both species trees reconstructed from the two methods, *P. suffruticosa* was placed as sister to Vitales, and they together formed a clade that was sister to Rosids. As the conflict between the concatenation‐based tree and coalescence‐based tree might indicate complicated evolutionary histories of genes in *K. fedtschenkoi*, we performed phylogenetic analyses excluding *K. fedtschenkoi* using the same two methods above. The resulting tree topologies (Supplementary Figure S4c,d) were congruent, supporting *P. suffruticosa* as sister to Vitales.

**Figure 2:**
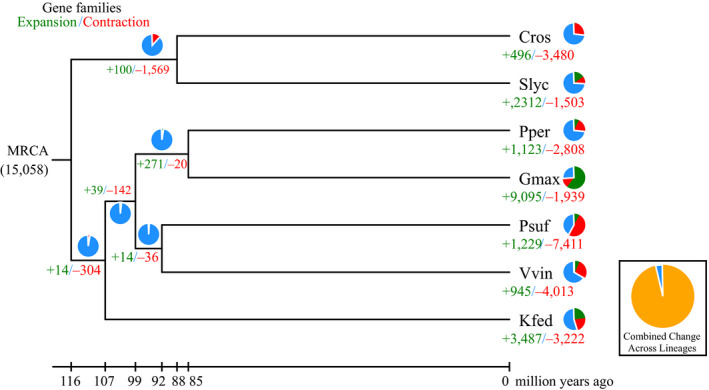
Phylogenetic tree showing divergence times and the evolution of gene family sizes. Pie graphs represent the proportion of gene families that expand and contract, with red for expansion and green for contraction. Cros: *Catharanthus roseus*, Slyc: *Solanum lycopersicum*, Pper: *Pyunus. persica*, Gmax: *Glycine max*, Psuf: *Paeonia suffruticosa*, Vvin: *Vitis vinifera*, Kfed: *Kalanchoë fedtschenkoi*

Based on the phylogenetic tree, *P. suffruticosa* was estimated to have separated from *V. vinifera* and *K. fedtschenkoi* approximately 92.3 and 98.9 Myr ago (Figure 2).The analysis of expansion and contraction of gene families between species using CAFÉ (De Bie et al., 2006) showed that 1,229 gene families were substantially expanded in *P. suffruticosa* and 7,411 gene families were contracted (Figure 2). The contraction is six times more than expansion, implying there are some missing genes due to the incomplete genome assembly or indicating that *P. suffruticosa* has undergone large‐scale gene loss events during the long domestication history.

### 3.4 Identification of MADS‐box genes from *P. suffruticosa* genome assembly and de novo transcriptome assembly

Using two methods of homology search of *Arabidopsis* MADS‐box proteins and HMMER search of MADS‐box domain profile, we identified 52 putative MADS‐box genes in *P. suffruticosa* genome assembly, including 36 type I and 16 type II genes (Table S13). Based on the phylogenetic analysis, 36 type I genes were divided into Mα and Mγ subgroups, which contained 19 and 17 members, respectively (Supplementary Figure S5). The Mβ MADS‐box genes are important in endosperm development (Masiero, Colombo, Grini, Schnittger, & Kater, 2011; Zhang et al., 2017), but are absent in our genome assembly. The number of type II MADS‐box genes in *P. suffruticosa* genome assembly are significantly reduced, compared with that in other eudicots, such as 32 in *Prunus mume*, 64 in *Populus trichocarpa,* and 47 in *Arabidopsis thaliana*. The missing of representative member of SVP, ANR1, FLC, and AGL13 classes in the 16 type II MADS‐box genes also indicated the incompleteness of the genome assembly.

In order to find potentially missing MADS‐box genes in *P. suffruticosa* genome, we performed de novo transcriptome assembly and obtained 40,179 nonredundant CDS sequences, from which 8 type I and 24 type II MADS‐box genes (Supplementary Table S14) were identified using the same methods described above. According to the phylogenetic tree, the missing MADS‐box genes of Mβ, SVP, ANR1, and AGL13 subfamilies in genome‐based prediction were recovered from transcriptome‐based prediction. No FLC orthologous gene was identified, indicating that this subfamily may have been lost in *P. suffruticosa*. In consideration of the possibility that the two MADS‐box gene sets may have overlaps, we used the 32 transcriptome‐derived MADS‐box genes as queries to BLASTP against the 52 genome‐derived MADS‐box genes. As listed in Supplementary Table S15, there were only seven pairs of genes showed high protein identity (>90%). If considering each of the seven pairs of genes to be identical, we obtained a total of 77 MADS‐box genes in *P. suffruticosa* in this study, including 44 type I and 33 type II genes. Of these type II genes, 18 were assigned to be ABCDE genes, including 5 A class genes, 5 B class genes, 2 C/D class genes, and 6 E class genes.

### 3.5 Expression of MADS‐box genes in different tissues

**Figure 3:**
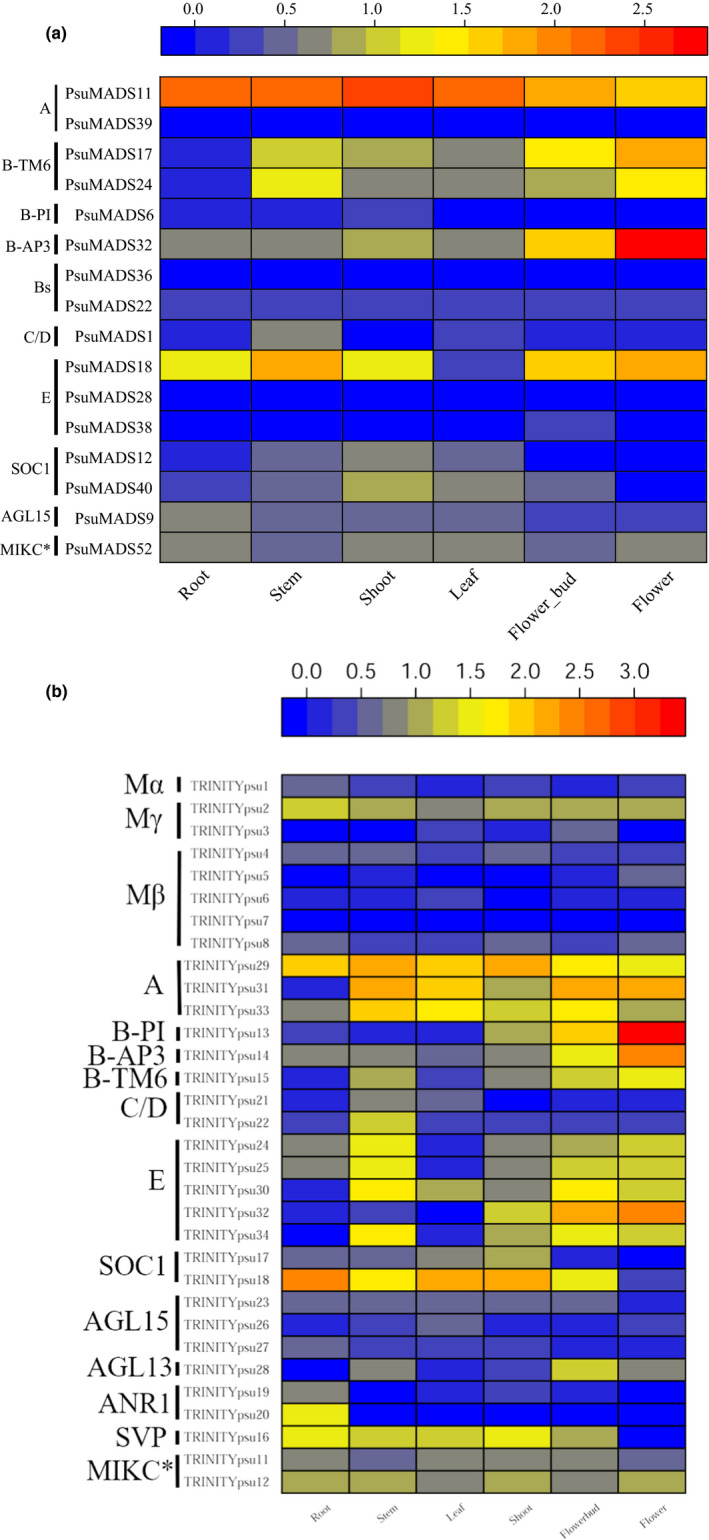
Expression profiles of MADS‐box genes in six organs. (a) Expression profiles of type II MADS‐box genes identified from the genome assembly. (b) Expression profiles of MADS‐box genes identified from the de novo transcriptome assembly. Transcriptome sequencing was employed to investigate expression patterns of MADS‐box genes. The *colour scale* shown at the top represents the normalized expression level (log_10_(FPKM + 1)). *Blue* indicates low expression levels while *red* indicates high levels

Transcriptome data showed that the expression of the type I MADS‐box genes, except PsuMADS5 and TRINITYpsu2, were all too weak to detect in the six different organs (Figure 3b). Since the six organs did not include the seed samples, we supposed that these type I genes might function in the seeds development as they do in *Arabidopsis* (Bouyer et al., 2011; Kang, Steffen, Portereiko, Lloyd, & Drews, 2008).

In contrast to the weak expression of type I genes, most of the type II genes had a moderate or high expression in certain tissues (Figure 3a,b). The SOC1 gene (TRINITYpsu18) was highly expressed in the vegetative tissues, in accordance with previous studies in tree peony (Zhang, Li, et al., 2015b). The SVP gene (TRINITYpsu16) was widely expressed in vegetative tissues and in flower bud, indicating that this gene may play multiple roles in tree peony development.

Most of the ABCDE genes exhibited the highest expressions in the flower bud and flower of *P. suffruticosa*, which conformed their important roles in flower development (Figure 3a,b). The A class genes (PsuMADS11, TRINITYpsu29, TRINITYpsu31, and TRINITYpsu33) were highly expressed in almost all six organs, implying they not only play the normal A class gene function in flower but also have multiple functions in other organs. Of the B class genes, AP3 gene (PsuMADS32 or TRINITYpsu14, they were nearly identical) and B‐PI gene (TRINITYpsu13) had similar expression profile in flower and flower bud, implying they act as heterodimers on the formation of petals and stamens as they do in *Arabidopsis* and in other core eudicots (Riechmann, Krizek, & Meyerowitz, 1996; Wuest et al., 2012). The expression of another PI gene (PsuMADS6) could not be detected in neither flower organ, implying this gene had lost the B class gene function. The two B‐TM6 genes (PsuMADS17 and PsuMADS24, and TRINITYpsu15 was nearly identical to PsuMADS17) had moderate to high expression in flower organs and stem, and weak expression in other three vegetative organs, which indicates that they may play multiple roles in tree peony. There were two C class genes (PsuMADS1 and TRINITYpsu22, and TRINITYpsu21 was nearly identical to PsuMADS1), which were homologous to *Arabidopsis* AGAMOUS (AG) gene (Supplementary Figure S6). Transcriptome data showed that the two C class genes were expressed at low level in flower organs. In the six E class genes, PsuMADS18 (TRINITYpsu24 and TRINITYpsu25 were nearly identical to PsuMADS18), TRINITYpsu30, TRINITYpsu32, and TRINITYpsu34 had moderate to high expression in flower organs, while PsuMADS28 and PsuMADS38 were barely expressed in any of the six organs.

## 4 DISCUSSION

The genome assembly of *Paeonia suffruticosa* presented in this study represents the largest genome sequenced in the dicots, to date. It is a big challenge to assemble so large genome with high ratio of heterozygosity and repetition. The draft genome was fragmentary and was far from completed. The most possible reason for the poor assembly is that most of the PacBio subreads, with an N50 of 14.5 kb and a mean length of 9.3kb, cannot span the repetitive regions across the large chromosome. Another reason for the poor assembly may be the PacBio data were insufficient. Although ~67× PacBio subreads were used, the error‐corrected data used for next assembling step in FALCON pipeline was about 20×. So, the key to improve the genome assembly of *Paeonia suffruticosa* is to get more and longer sequencing reads, which should be taken into account in improvement of this genome assembly in future.

The draft genome of *P. suffruticosa* is the first genome sequenced for the Paeoniaceae, and the second sequenced species in Saxifragales. The coalescent phylogenetic tree (Supplementary Figure S4 (a)) placed the two sequenced species of Saxifragales into two different clades, implying paraphyly of the order of Saxifragales. According to this coalescent tree, the position of *P. suffruticosa* supports a relationship of ((Saxifragales and Vitales) and Rosids) and the position of *K. fedtschenkoi* supports a relationship of ((Rosids and Vitales) and Saxifragales). But the monophyly of Saxifragales has been strongly supported by molecular data in previous studies (Soltis et al., 2013; Soltis, Soltis, Endress, & Chase, 2005). Moreover, the proportion of gene trees that supported the tree topologies at each node showed that the positions of *P. suffruticosa* and *K. fedtschenkoi* in the coalescent tree were both weakly supported. So, we can reject neither of the possible position of Saxifragales in the phylogenetic tree. Although a “superrosid” clade of Saxifragales, Vitales, and Rosids is strongly supported, the relationships among these three groups have been debated for many years and different topologies were proposed by several studies based on plastid and nuclear genomes (Moore et al., 2011; Moore, Soltis, Bell, Burleigh, & Soltis, 2010; Soltis et al., 2011; Zeng et al., 2017). It is believed that the challenge of resolving the relationships among the three groups is ascribed to the rapid diversification of early eudicots (Magallon, Gomez‐Acevedo, Sanchez‐Reyes, & Hernandez‐Hernandez, 2015; Moore et al., 2010) and the concomitant incomplete lineage sorting in phylogenetic tree reconstructing process (Degnan & Rosenberg, 2009). In previous studies, the split between Vitaceae and Saxifragales was dated to 112–101 mya based on dataset of plastid genes (Moore et al., 2010), and the split between Saxifragales and Rosids was dated to *c.* 112.4 mya based on dataset of nuclear genes (Zeng et al., 2017), indicating early and rapid diversification in superrosids. Thus, a better resolution among the three groups of superrosids needs further and more extensive genomic and taxon sampling, especially of Saxifragales species.

Tree peonies have an extensive history of domestication, during which the most important traits that have changed are the shapes and colors of the flowers, especially the number and colors of the petals. Most of the tree peony cultivars have various number of whorls of petals because of stamen petalody, but the inherent mechanism responsible for stamen or petal development in tree peony is still unclear. MADS‐box transcription factors play important roles in plant development, especially in flower development. In the classical ABC(E) model of floral organ identities, A, B, C, and E all encode members of the MADS‐box TF family, with A class genes specifying sepals, A + B + E specifying petals, B + C + E specifying stamens, and C + E specifying carpels (Litt & Kramer, 2010; Theissen & Saedler, 2001; Wellmer, Graciet, & Riechmann, 2014). According to the ABCDE model of flower development, B and C class MADS‐box genes determined the identity of petal and stamen. In this study, we identified five nonredundant B class genes and two C class genes from the combined dataset of genome assembly and transcriptome assembly. Except one B‐PI gene, the other four B class genes were all highly expressed in flower. It was worth noting that the sequences of the two B‐TM6 genes had been proved to be different among wild species and cultivars of tree peonies, which was explained to be related to stamen petalody and different flower shape formation in tree peonies (Shu et al., 2012). In the B class genes, TM6 are paralogs of AP3. The function of TM6 has been mostly studied in asterid model plant, such as petunia and tomato, in which TM6 orthologs are mainly expressed in stamens and carpels and may function redundantly in stamen identity with AP3 (Gemma, Pan, Emmanuel, Levy, & Irish, 2006; Rijpkema et al., 2006). So, it means TM6 may have part of C class gene function in spite of its belonging to B class. Meanwhile, the expression of C class genes in this study was very low in flower organs. In *Arabidopsis*, mutations in the AG gene result in the double flower phenotype, that is, the stamens are replaced by petals and carpels are replaced by a new flower (Yanofsky et al., 1990). The loss‐of‐function or restricted expression of the C function genes has shown to play a central role in the production of excessive numbers of petals in many different species, such as in *Thalictrum thalictroides* (Galimba et al., 2012), *Prunus lannesiana* (Liu, Zhang, Liu, Li, & Lu, 2013), *Camellia japonica* (Sun et al., 2014), and in rose (Dubois et al., 2010). During plant domestication, the causal mutations for convergent changes in key traits are likely to be located in particular genes (Lenser & Theissen, 2013). So, we suppose that the restriction of C class gene function may be responsible for the stamen petalody in tree peony cultivars. In addition, when the function of C class genes is restricted, the expression of TM6 genes can assure the normal development of carpels. It is reasonable to infer that the combined activity of AP3/PI and TM6 genes determines the formation of petal and stamen as well as the conversion between them when the C class gene function is restricted.

## 5 CONCLUSION

This study presents the first genome in the family Paeoniaceae and the largest eudicots genome sequenced to date. This genome is also an important addition to Saxifragales genomic resources, which will facilitate the research of the phylogeny of this highly diverse clade. By integrating this genome with transcriptome data, we have demonstrated the use of this genome to explore the molecular mechanism underlying the flower development specified in this ornamental plant and suggested a modified BC model in the formation of petal and stamen. It can be expected that this genome will aid in deciphering the formation of specific and important traits in tree peony, such as various flower colors, oil accumulation in seeds, biosynthesis pathways of pharmacologically active metabolites, and adaption under domestication.

## AUTHORS’ CONTRIBUTIONS

Canjun Zhang, Jiangtao Huang, Yang Dong, and Gengyun Zhang conceived and designed the project. Shuzuo lv, Zhanying Wang, Erqiang Wang, and Xiaogai Hou collected the plant materials and performed the experiments. Bing Yang, Shu Cheng, Gang Huang, Jie Wang, Shiming Li, Xin Jin, Lei Lan, Kang Yu, Ning Li, and Xuemei Ni analyzed the data. Shu Cheng, Shuzuo Lv, Gengyun Zhang, and Canjun Zhang wrote the paper. All authors read and consented to the final version of the manuscript.

## Data Availability

All raw sequencing data, genome assembly sequences and gene annotations are available at the China National GeneBank (CNGB) Nucleotide Sequence Archive (CNSA) under BioProject accession code CNP0000281. The genome sequencing reads and genome assembly are deposited in CNSA with the Biosample accession CNS0044072. The RNA‐seq reads are deposited in CNSA with the BioSample accession codes CNS0044073—CNS0044078.
