# Supplementary material for: Draft genome of the famous ornamental plant Paeonia suffruticosa
Source: Ecol Evol. 2020 May 12;10(11):4518–30. doi: 10.1002/ece3.5965 (PMC7297784; doi:10.1002/ece3.5965)
Supplement: Supplementary file 1 [file ECE3-10-4518-s001.doc]

**Supplementary Table 1. Statistics of RNA sequencing data generated by BGISEQ-500.**

| Tissue | Read length(bp) | Raw data | | High-quality data | |
| --- | --- | --- | --- | --- | --- |
|  |  | Total bases | Q20  (%) | Total bases | Q20  (%) |
|  |  | (Gb) |  | (Gb) |  |
| Root | 100 | 7.3 | 96.14 | 7.18 | 96.25 |
| Stem | 100 | 7.26 | 96.74 | 7.15 | 96.82 |
| Shoot | 100 | 7.8 | 96.62 | 7.7 | 96.71 |
| Leaf | 100 | 6.93 | 96.62 | 6.85 | 96.76 |
| Flower | 100 | 8.58 | 96.65 | 8.46 | 96.73 |
| Flower bud | 100 | 7.84 | 96.68 | 7.75 | 96.75 |

**Supplementary Table 2. Statistics of genome size estimation using jellyfish for 17, 19, 21 and 23 kmers.**

| Genome | K-mer length (bp) | K-mer numbers | K-mer depths | Estimated genome size (bp) | Reads numbers | Genome coverage |
| --- | --- | --- | --- | --- | --- | --- |
| *Paeonia suffruticosa* | 17 | 519,130,610,870 | 38 | 13,661,331,865 | 6,420,894,334 | 46.53 |
|  | 19 | 502,261,953,445 | 35 | 14,350,341,527 | 6,420,894,334 | 44.30 |
|  | 21 | 487,481,820,870 | 33 | 14,772,176,390 | 6,420,894,334 | 43.03 |
|  | 23 | 472,710,263,910 | 30 | 15,757,008,797 | 6,420,894,334 | 40.34 |

The genome size was estimated according to the formula Genome size = k-mer numbers/k-mer depths

**Supplementary Table 3. Comparison of different parameters used in seven genome assembly versions.**

| Parameters | Assembly version 1 | Assembly version 2 | Assembly version 3 | Assembly version 4 | Assembly version 5 | Assembly version 6 | Assembly version 7 |
| --- | --- | --- | --- | --- | --- | --- | --- |
| length_cutoff_pr | 9000 | 9000 | 9000 | 9000 | 9000 | 6000 | 6000 |
| pa_HPCdaligner_option | -v -B286 -t12 -w8 -M24 -e.75 -k18 -h380 -l2800 -s1000 -T4 | -v -B286 -t12 -w8 -M24 -e.75 -k18 -h380 -l2800 -s1000 -T4 | -v -B286 -t12 -w8 -M24 -e.75 -k18 -h380 -l2800 -s1000 -T4 | -v -B286 -t12 -w8 -M24 -e.75 -k18 -h380 -l2800 -s1000 -T4 | -v -B286 -t12 -w8 -M24 -e.75 -k18 -h380 -l2800 -s1000 -T4 | -v -B286 -t12 -w8 -M24 -e.75 -k18 -h380 -l2800 -s1000 -T4 | -v -B286 -t12 -w8 -M24 -e.75 -k18 -h380 -l2800 -s1000 -T4 |
| ovlp_HPCdaligner_option | -v -B180 -t12 -k18 -h180 -e.95 -l2000 -s1000 | -v -B180 -t12 -k18 -h180 -e.95 -l2000 -s1000 | -v -B180 -t12 -k18 -h180 -e.95 -l2000 -s1000 | -v -B180 -t12 -k18 -h180 -e.95 -l2200 -s1000 | -v -B180 -t12 -k18 -h180 -e.95 -l2200 -s1000 | -v -B256 -t12 -k18 -h180 -e.95 -l1800 -s1000 | -v -B256 -t12 -k18 -h180 -e.95 -l1800 -s1000 |
| overlap_filtering_setting | --max_diff 40 --max_cov 60, --min_cov 1 | --max_diff 40 --max_cov 60, --min_cov 2 | --max_diff 60 --max_cov 60, --min_cov 3 | --max_diff 60 --max_cov 60, --min_cov 2 | --max_diff 60 --max_cov 60, --min_cov 1 | --max_diff 60 --max_cov 60, --min_cov 2 | --max_diff 60 --max_cov 60, --min_cov 3 |
| N50 (bp) | 76724 | 66650 | 50731 | 54677 | 73851 | 49745 | 48400 |

**Supplementary Table 4. Comparison of seven genome assembly versions with different assembly parameters.**

|  | Assembly version 1 | Assembly version 2 | Assembly version 3 | Assembly version 4 | Assembly version 5 | Assembly version 6 | Assembly version 7 |
| --- | --- | --- | --- | --- | --- | --- | --- |
| Sequences | 274,501 | 279,192 | 421,576 | 415,962 | 279,192 | 499,789 | 480,520 |
| Total length of sequences | 11,771,671,418 | 11,521,834,671 | 12,266,303,260 | 12,964,260,471 | 11,521,834,671 | 13,776,032,646 | 13,157,150,223 |
| Longest sequence length | 898,840 | 957,532 | 993,863 | 993,926 | 898,840 | 729,183 | 872,000 |
| N50 | 76,724 | 66,650 | 50,731 | 54,677 | 73,851 | 49,745 | 48,400 |
| Sequences (>10k) | 218,983 | 219,670 | 238,750 | 304,728 | 219,670 | 339,893 | 328,237 |
| Sequences (>5k) | 250,273 | 253,320 | 281,161 | 370,949 | 253,320 | 427,546 | 414,214 |
| Sequences (>1k) | 271,186 | 275,686 | 309,091 | 410,735 | 275,686 | 489,583 | 472,023 |
| Sequences (>100 bp) | 274,501 | 279,192 | 313,552 | 415,962 | 279,192 | 499,789 | 480,520 |

**Supplementary Table 5. BUSCO analysis results of eight genome assembly versions.**

|  | V 1 | V2 | V3 | V4 | V5 | V6 | V7 | V_final |
| --- | --- | --- | --- | --- | --- | --- | --- | --- |
| Complete BUSCOs | 840  (58.3%) | 832  (57.8%) | 828  (57.5%) | 845  (58.7%) | 830  (57.6%) | 881  (61.2%) | 857  (59.5%) | 952  (66.1%) |
| Complete Single-Copy BUSCOs | 620  (43.1%) | 621  (43.1%) | 625  (43.4%) | 613  (42.6%) | 627  (43.5%) | 612  (42.5%) | 619  (43%) | 855  (59.4%) |
| Complete Duplicated BUSCOs | 220  (15.3%) | 211  (14.7%) | 203  (14.1%) | 232  (16.1%) | 203  (14.1%) | 269  (18.7%) | 238  (16.5%) | 97  (6.7) |
| Fragmented BUSCOs | 105  (7.3%) | 109  (7.6%) | 90  (6.2%) | 101  (7%) | 99  (6.9%) | 110  (7.6%) | 96  (6.7%) | 80  (5.6%) |
| Missing BUSCOs | 495  (34.4%) | 499  (34.7%) | 522  (36.2%) | 494  (34.3%) | 511  (35.5%) | 449  (31.2%) | 487  (33.8%) | 408  (28.3%) |
| Total BUSCO groups searched | 1440 | 1440 | 1440 | 1440 | 1440 | 1440 | 1440 | 1440 |

**Supplementary Table 6. Statistics of the mapping ratio of RNA sequencing reads.**

| Tissue | Number of input reads | Number of mapped reads | Percentage | Data source |
| --- | --- | --- | --- | --- |
| Root | 71806434 | 52962863 | 73.76% | This study |
| Stem | 71548766 | 52942689 | 74.00% | This study |
| Shoot | 77021514 | 56224770 | 73.00% | This study |
| Leaf | 68475402 | 49424074 | 72.18% | This study |
| Flower | 84641188 | 61883907 | 73.11% | This study |
| Flower bud | 77494346 | 56493832 | 72.90% | This study |
| Petal | 53763848 | 23800 | 0.04% |  |
| Flower bud | 53814286 | 35400563 | 65.78% |  |
| Flower | 63567136 | 41794387 | 65.75% |  |
| Leaf | 401547112 | 274679351 | 68.41% |  |

**Supplementary Table 7**. Repeat statistics

| **Repeat Element** | **Length (bp)** | **% of sequence** |
| --- | --- | --- |
| **SINEs** | 157,802,455 | 1.15 |
|  |  |  |
| **LINEs** | 1,931,149,253 | 14.02 |
|  |  |  |
| **LTR elements**  **Copia**  **Gspsy** | 6,874,219,419  704,703,090  5,360,466,141 | 49.90  5.12  38.91 |
|  |  |  |
| **DNA elements** | 1,861,990,994 | 13.52 |
|  |  |  |
| **Helitron** | 56,007,488 | 0.41 |
|  |  |  |
| **Unclassified** | 2,058,919,316 | 14.95 |
|  |  |  |
| **Satellites**  **Simple repeats** | 605,358  882,320,378 | 0.00  6.40 |
|  |  |  |
| **Total Repeats** | 11,054,226,421 | 80.24 |

**Supplementary Table 8.** Gene model annotation

| **Gene set** | | **Number** | **Average transcript length (bp)** | **Average CDS length (bp)** | **Average exon per gene** | **Average exon length (bp)** | **Average intron length (bp)** |
| --- | --- | --- | --- | --- | --- | --- | --- |
| ***De novo*** | AUGUSTUS | 111467 | 5011 | 1023 | 5.14 | 199 | 963 |
| **Homolog** | *Glycine max* | 36721 | 6521 | 1062 | 5.81 | 183 | 1136 |
|  | *S. lycopersicon* | 31942 | 6226 | 843 | 4.63 | 182 | 1484 |
|  | *V. vinifera* | 26447 | 6582 | 929 | 5.49 | 169 | 1258 |
|  | *A. thaliana* | 15907 | 6135 | 1016 | 5.30 | 192 | 1191 |
|  | *P. persica* | 23992 | 6869 | 1089 | 5.69 | 191 | 1233 |
| **RNA-Seq** | | 55788 | 9768 | 1356 | 4.16 | 326 | 2664 |
| **Final set** | | 35687 | 6747 | 1188 | 5.66 | 210 | 1192 |

**Supplementary Table 9. Comparison of gene metrics for the genomes of *P. suffruticosa* and six other plants**

| **spiecies** | **Gene**  **number** | **Average transcript length (bp)** | **Average CDS length (bp)** | **Average exon per gene** | **Average exon length (bp)** | **Average intron length (bp)** |
| --- | --- | --- | --- | --- | --- | --- |
| ***P. suffruticosa*** | 35687 | 6747 | 1188 | 5.66 | 210 | 1192 |
| ***S. lycopersicum*** | 35768 | 4010 | 1027 | 4.67 | 220 | 597 |
| ***A. thaliana*** | 27416 | 1869 | 1218 | 5.13 | 238 | 158 |
| ***G. max*** | 42909 | 3317 | 1262 | 5.82 | 217 | 426 |
| ***V. vinifera*** | 25395 | 6116 | 1175 | 6.1 | 193 | 968 |
| ***P. persica*** | 27837 | 2446 | 1215 | 4.91 | 248 | 315 |
| ***K. fedtschenkoi*** | 30964 | 2211 | 1129 | 4.88 | 231 | 279 |

**Supplementary Table 10. Gene function annotation**

|  | | **Number** | **Percent (%)** |
| --- | --- | --- | --- |
| **Total** | | 35,687 |  |
| **Annotated** | InterPro | 31,885 | 89.35 |
|  | GO | 19,500 | 54.64 |
|  | KEGG | 23,804 | 66.70 |
|  | COG | 12,387 | 34.71 |
|  | Swissprot | 32,258 | 90.39 |
|  | Nr | 28,861 | 80.87 |
|  | TrEMBL | 34,498 | 96.67 |
| **Unannotated** | | 833 | 2.33 |

**Supplementary Table 11. Non-coding RNA genes in the *P. suffruticosa* genome**

| Type | | Copy | Average length (bp) | Total length (bp) | % of genome |
| --- | --- | --- | --- | --- | --- |
| miRNA | | 960 | 91.75 | 88080 | 0.000639 |
| tRNA | | 12154 | 75.47 | 917266 | 0.006658 |
| rRNA |  | 15108 | 204.32 | 3086874 | 0.022408 |
|  | 18S | 1285 | 1321.91 | 1698657 | 0.012330 |
|  | 28S | 3404 | 135.49 | 461205 | 0.003348 |
|  | 5.8S | 932 | 153.71 | 143254 | 0.001040 |
|  | 5S | 9487 | 82.61 | 783758 | 0.005689 |
| snRNA |  | 1055 | 120.69 | 127331 | 0.000924 |
|  | CD-box | 452 | 110.68 | 50026 | 0.000363 |
|  | HACA-box | 82 | 133.29 | 10930 | 0.000079 |
|  | splicing | 497 | 130.28 | 64751 | 0.000470 |

**Supplementary Table 12. Orthology analysis at the protein level of predicted genes in *P. suffruticosa* with those annotated in other seven plant species**

| Species | Total predicted  genes | Genes in othologous  families | Genes not in othologous families | No. of othologous families | Species-  specific familes | Average No. of genes per family |
| --- | --- | --- | --- | --- | --- | --- |
| *P. suffruticosa* | 35687 | 22279 | 13408 | 10882 | 1794 | 2.05 |
| *G. max* | 42859 | 38668 | 4191 | 13998 | 1063 | 2.76 |
| *O. sativa* | 38964 | 27053 | 11911 | 13042 | 2098 | 2.07 |
| *P. persica* | 27792 | 24428 | 3364 | 13737 | 499 | 1.78 |
| *V. vinifera* | 25329 | 19382 | 5947 | 12794 | 605 | 1.51 |
| *S. lycopersicum* | 33585 | 25950 | 7635 | 13970 | 940 | 1.86 |
| *K. fedtschenkoi* | 30964 | 24874 | 6090 | 13312 | 972 | 1.87 |
| *C. roseus* | 34363 | 30037 | 4326 | 12816 | 629 | 2.34 |

**Supplementary Table 13. List of 52 MADS-box genes identified in *P. suffruticosa* genome assembly**.

| Gene ID | Gene name | Protein(aa) | Type | subfamily |
| --- | --- | --- | --- | --- |
| psu.T.00033243.1 | PsuMADS44 | 230 | Type I | Mα |
| psu.T.00014444.1 | PsuMADS16 | 166 | Type I | Mα |
| psu.T.00035357.1 | PsuMADS50 | 195 | Type I | Mα |
| psu.T.00017855.1 | PsuMADS21 | 192 | Type I | Mα |
| psu.T.00018280.1 | PsuMADS23 | 223 | Type I | Mα |
| psu.T.00014419.1 | PsuMADS15 | 266 | Type I | Mα |
| psu.T.00022121.1 | PsuMADS29 | 241 | Type I | Mα |
| psu.T.00025838.1 | PsuMADS33 | 266 | Type I | Mα |
| psu.T.00032332.1 | PsuMADS42 | 279 | Type I | Mα |
| psu.T.00035533.1 | PsuMADS51 | 266 | Type I | Mα |
| psu.T.00032415.1 | PsuMADS43 | 216 | Type I | Mα |
| psu.T.00017839.1 | PsuMADS20 | 216 | Type I | Mα |
| psu.T.00034999.1 | PsuMADS46 | 216 | Type I | Mα |
| psu.T.00005099.1 | PsuMADS4 | 97 | Type I | Mα |
| psu.T.00003613.1 | PsuMADS3 | 151 | Type I | Mα |
| psu.T.00034475.1 | PsuMADS45 | 248 | Type I | Mα |
| psu.T.00014380.1 | PsuMADS14 | 465 | Type I | Mα |
| psu.T.00035312.1 | PsuMADS49 | 155 | Type I | Mα |
| psu.T.00008326.1 | PsuMADS7 | 350 | Type I | Mα |
| psu.T.00005107.1 | PsuMADS5 | 869 | Type I | Mγ |
| psu.T.00032173.1 | PsuMADS41 | 270 | Type I | Mγ |
| psu.T.00011759.1 | PsuMADS10 | 170 | Type I | Mγ |
| psu.T.00035026.1 | PsuMADS47 | 220 | Type I | Mγ |
| psu.T.00017670.1 | PsuMADS19 | 241 | Type I | Mγ |
| psu.T.00002273.1 | PsuMADS2 | 217 | Type I | Mγ |
| psu.T.00023811.1 | PsuMADS30 | 217 | Type I | Mγ |
| psu.T.00021135.1 | PsuMADS26 | 234 | Type I | Mγ |
| psu.T.00013739.1 | PsuMADS13 | 165 | Type I | Mγ |
| psu.T.00029383.1 | PsuMADS35 | 165 | Type I | Mγ |
| psu.T.00021861.1 | PsuMADS27 | 165 | Type I | Mγ |
| psu.T.00031080.1 | PsuMADS37 | 184 | Type I | Mγ |
| psu.T.00028490.1 | PsuMADS34 | 197 | Type I | Mγ |
| psu.T.00025172.1 | PsuMADS31 | 284 | Type I | Mγ |
| psu.T.00035042.1 | PsuMADS48 | 137 | Type I | Mγ |
| psu.T.00009064.1 | PsuMADS8 | 186 | Type I | Mγ |
| psu.T.00020112.1 | PsuMADS25 | 128 | Type I | Mγ |
| psu.T.00014484.1 | PsuMADS17 | 287 | MIKC^c^ | B-TM6 |
| psu.T.00019151.1 | PsuMADS24 | 200 | MIKC^c^ | B-TM6 |
| psu.T.00007312.1 | PsuMADS6 | 161 | MIKC^c^ | B-PI |
| psu.T.00025444.1 | PsuMADS32 | 202 | MIKC^c^ | B-AP3 |
| psu.T.00029581.1 | PsuMADS36 | 180 | MIKC^c^ | Bs |
| psu.T.00018021.1 | PsuMADS22 | 239 | MIKC^c^ | Bs |
| psu.T.00001030.1 | PsuMADS1 | 221 | MIKC^c^ | C/D |
| psu.T.00016071.1 | PsuMADS18 | 245 | MIKC^c^ | E |
| psu.T.00021870.1 | PsuMADS28 | 109 | MIKC^c^ | E |
| psu.T.00031511.1 | PsuMADS38 | 96 | MIKC^c^ | E |
| psu.T.00013683.1 | PsuMADS12 | 185 | MIKC^c^ | SOC1 |
| psu.T.00031880.1 | PsuMADS40 | 257 | MIKC^c^ | SOC1 |
| psu.T.00009401.1 | PsuMADS9 | 292 | MIKC^c^ | AGL15 |
| psu.T.00035667.1 | PsuMADS52 | 130 | MIKC^*^ |  |
| psu.T.00013212.1 | PsuMADS11 | 2299 | MIKC^c^ | A |
| psu.T.00031771.1 | PsuMADS39 | 239 | MIKC^c^ | A |

| **Supplementary Table 14. List of 32 MADS-box genes identified from de novo transcriptome assembly.** | | | | |
| --- | --- | --- | --- | --- |
| **Gene ID** | **Gene name** | **Protein(aa)** | **Type** | **subfamily** |
| root@TRINITY_DN19727_c0_g1_i1_p1 | TRINITYpsu1 | 174 | Type1 | Mα |
| flowerbud@TRINITY_DN15055_c0_g1_i2_p1 | TRINITYpsu2 | 286 | Type1 | Mγ |
| flowerbud@TRINITY_DN8964_c0_g1_i1_p1 | TRINITYpsu3 | 214 | Type1 | Mγ |
| root@TRINITY_DN55115_c0_g1_i1_p1 | TRINITYpsu4 | 263 | Type1 | Mβ |
| flower@TRINITY_DN35146_c0_g1_i1_p1 | TRINITYpsu5 | 202 | Type1 | Mβ |
| leaf@TRINITY_DN12952_c0_g1_i2_p1 | TRINITYpsu6 | 226 | Type1 | Mβ |
| leaf@TRINITY_DN12952_c0_g1_i1_p1 | TRINITYpsu7 | 196 | Type1 | Mβ |
| shoot@TRINITY_DN25785_c2_g4_i1_p1 | TRINITYpsu8 | 206 | Type1 | Mβ |
| flowerbud@TRINITY_DN19091_c1_g1_i4_p1 | TRINITYpsu29 | 255 | Type2 | A |
| flowerbud@TRINITY_DN21938_c2_g3_i1_p1 | TRINITYpsu31 | 243 | Type2 | A |
| stem@TRINITY_DN20517_c9_g1_i11_p1 | TRINITYpsu33 | 283 | Type2 | A |
| flowerbud@TRINITY_DN21152_c1_g4_i9_p1 | TRINITYpsu13 | 213 | Type2 | B-PI |
| flowerbud@TRINITY_DN15567_c0_g1_i1_p1 | TRINITYpsu14 | 209 | Type2 | B-AP3 |
| stem@TRINITY_DN19828_c0_g1_i1_p1 | TRINITYpsu15 | 271 | Type2 | B-TM6 |
| stem@TRINITY_DN9007_c0_g1_i1_p1 | TRINITYpsu21 | 242 | Type2 | C/D |
| stem@TRINITY_DN21626_c3_g2_i11_p1 | TRINITYpsu22 | 259 | Type2 | C/D |
| leaf@TRINITY_DN5630_c0_g1_i1_p1 | TRINITYpsu24 | 199 | Type2 | E |
| flowerbud@TRINITY_DN20463_c3_g2_i5_p1 | TRINITYpsu25 | 270 | Type2 | E |
| flowerbud@TRINITY_DN11607_c0_g1_i1_p1 | TRINITYpsu30 | 244 | Type2 | E |
| flowerbud@TRINITY_DN20493_c0_g1_i1_p1 | TRINITYpsu32 | 244 | Type2 | E |
| flowerbud@TRINITY_DN20735_c0_g2_i1_p1 | TRINITYpsu34 | 245 | Type2 | E |
| shoot@TRINITY_DN24333_c0_g2_i4_p1 | TRINITYpsu17 | 203 | Type2 | SOC1 |
| flowerbud@TRINITY_DN21796_c1_g1_i2_p1 | TRINITYpsu18 | 226 | Type2 | SOC1 |
| leaf@TRINITY_DN11360_c0_g1_i1_p1 | TRINITYpsu23 | 290 | Type2 | AGL15 |
| flower@TRINITY_DN53055_c0_g1_i1_p1 | TRINITYpsu26 | 179 | Type2 | AGL15 |
| root@TRINITY_DN21605_c1_g1_i1_p1 | TRINITYpsu27 | 270 | Type2 | AGL15 |
| flowerbud@TRINITY_DN20107_c6_g2_i5_p1 | TRINITYpsu28 | 275 | Type2 | AGL13 |
| root@TRINITY_DN27149_c1_g2_i9_p1 | TRINITYpsu19 | 262 | Type2 | ANR1 |
| root@TRINITY_DN27149_c1_g7_i6_p1 | TRINITYpsu20 | 238 | Type2 | ANR1 |
| leaf@TRINITY_DN16737_c0_g1_i3_p1 | TRINITYpsu16 | 230 | Type2 | SVP |
| flowerbud@TRINITY_DN18891_c0_g1_i1_p1 | TRINITYpsu11 | 353 | Type2 | MIKC* |
| leaf@TRINITY_DN20160_c0_g2_i10_p1 | TRINITYpsu12 | 366 | Type2 | MIKC* |

**Supplementary Table 15. Statistics of protein identity for two datasets of MADS-box genes identified from genome assembly and de no transcriptome assembly.**

| **subfamily** | **query_id** | **target_id** | **identity** | **E-value** |
| --- | --- | --- | --- | --- |
| Mβ | TRINITYpsu4 | PsuMADS5 | 26.446 | 8.79E-06 |
| Mγ | TRINITYpsu3 | PsuMADS41 | 86 | 8.30E-126 |
| Mα | TRINITYpsu1 | PsuMADS45 | 69.461 | 3.74E-74 |
| Mγ | TRINITYpsu2 | PsuMADS41 | 43.721 | 2.68E-54 |
| AGL15 | TRINITYpsu27 | PsuMADS9 | 97.026 | 0 |
| C/D | TRINITYpsu21 | PsuMADS1 | 93.778 | 1.62E-159 |
| AGL15 | TRINITYpsu23 | PsuMADS9 | 55.392 | 1.99E-71 |
| A | TRINITYpsu29 | PsuMADS18 | 47.541 | 4.89E-53 |
| ANR1 | TRINITYpsu20 | PsuMADS1 | 44.886 | 2.07E-46 |
| AGL13 | TRINITYpsu28 | PsuMADS18 | 39.721 | 4.30E-56 |
| E | TRINITYpsu30 | PsuMADS18 | 50.607 | 6.70E-80 |
| B-TM6 | TRINITYpsu15 | PsuMADS17 | 94.902 | 0 |
| E | TRINITYpsu24 | PsuMADS18 | 100 | 8.66E-128 |
| A | TRINITYpsu33 | PsuMADS18 | 42.857 | 3.35E-52 |
| SVP | TRINITYpsu16 | PsuMADS9 | 36.975 | 1.36E-39 |
| AGL15 | TRINITYpsu26 | PsuMADS9 | 60.87 | 2.39E-74 |
| B-AP3 | TRINITYpsu14 | PsuMADS32 | 99.507 | 3.78E-154 |
| MIKC* | TRINITYpsu11 | PsuMADS52 | 78.652 | 7.43E-48 |
| SOC1 | TRINITYpsu17 | PsuMADS40 | 80.597 | 7.56E-111 |
| B-PI | TRINITYpsu13 | PsuMADS6 | 67.883 | 1.30E-66 |
| A | TRINITYpsu31 | PsuMADS18 | 48.438 | 4.36E-56 |
| E | TRINITYpsu25 | PsuMADS18 | 90.706 | 0 |
| E | TRINITYpsu32 | PsuMADS18 | 60.246 | 6.72E-103 |
| C/D | TRINITYpsu22 | PsuMADS1 | 66.667 | 1.72E-104 |
| ANR1 | TRINITYpsu19 | PsuMADS1 | 52.817 | 6.36E-49 |
| E | TRINITYpsu34 | PsuMADS18 | 50.202 | 9.35E-78 |
| SOC1 | TRINITYpsu18 | PsuMADS40 | 46.226 | 4.00E-49 |
| MIKC* | TRINITYpsu12 | PsuMADS52 | 98.876 | 1.10E-59 |


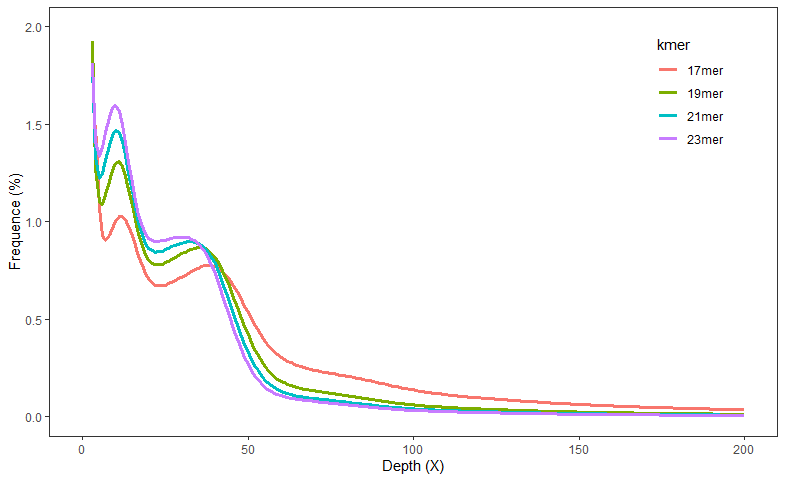


**Supplementary Fig. 1 kmer analysis of the *P. suffruticosa* genome*.*** According to the kmer depth distribution curves, we estimate that the genome size of *P. suffruticosa* is approximately 13.66-15.76 Gb.


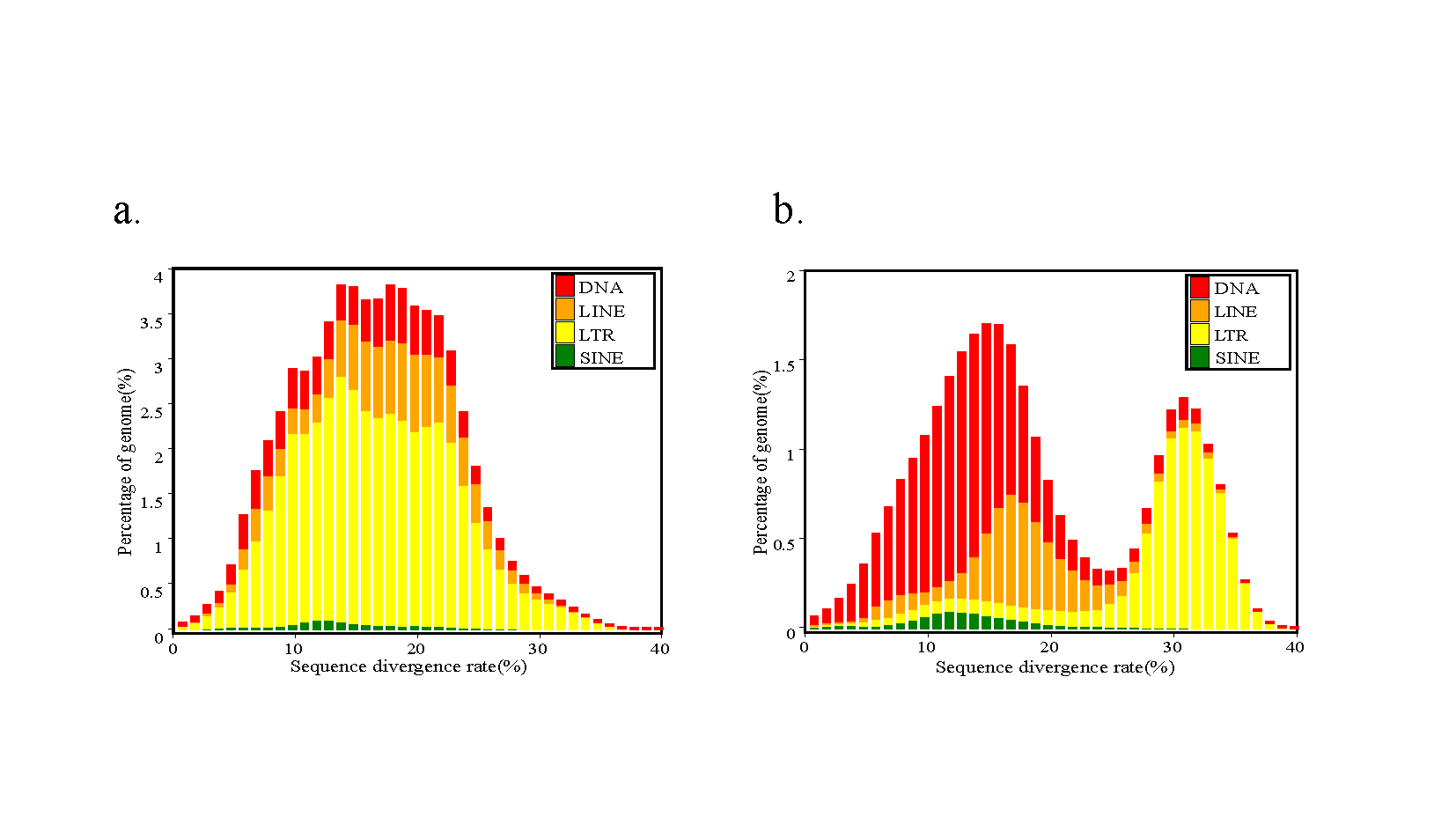


**Supplementary Fig. 2** **The distribution of sequence divergence rates of TEs in the *P. suffruticosa* genome. (a)**Based on the Repbase-comparison approach; **(b)**Based on the *de novo* approach.


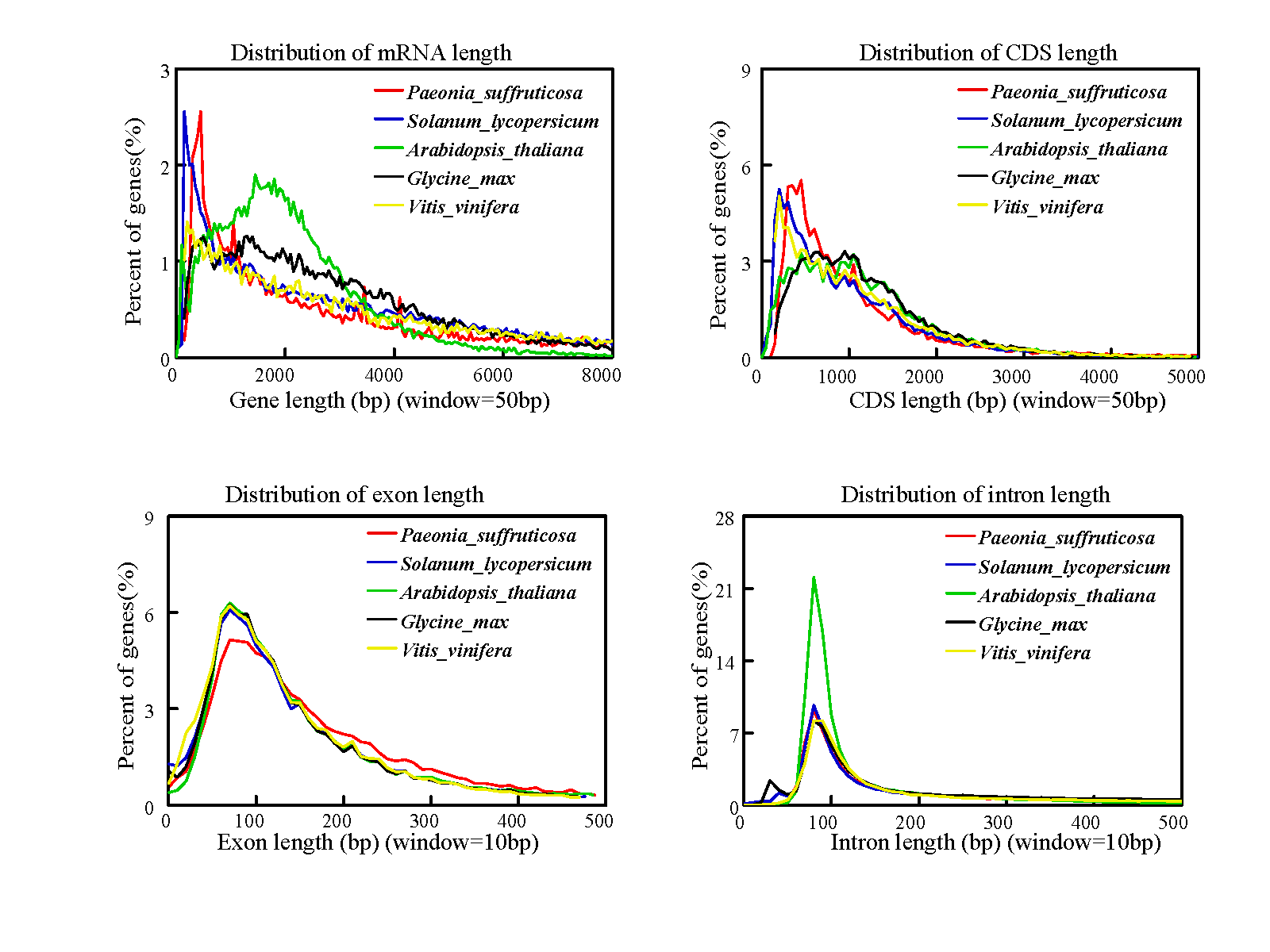


**Supplementary Fig. 3 Comparison of the length distribution of gene sets in *P. suffruticosa* and four other plants.**


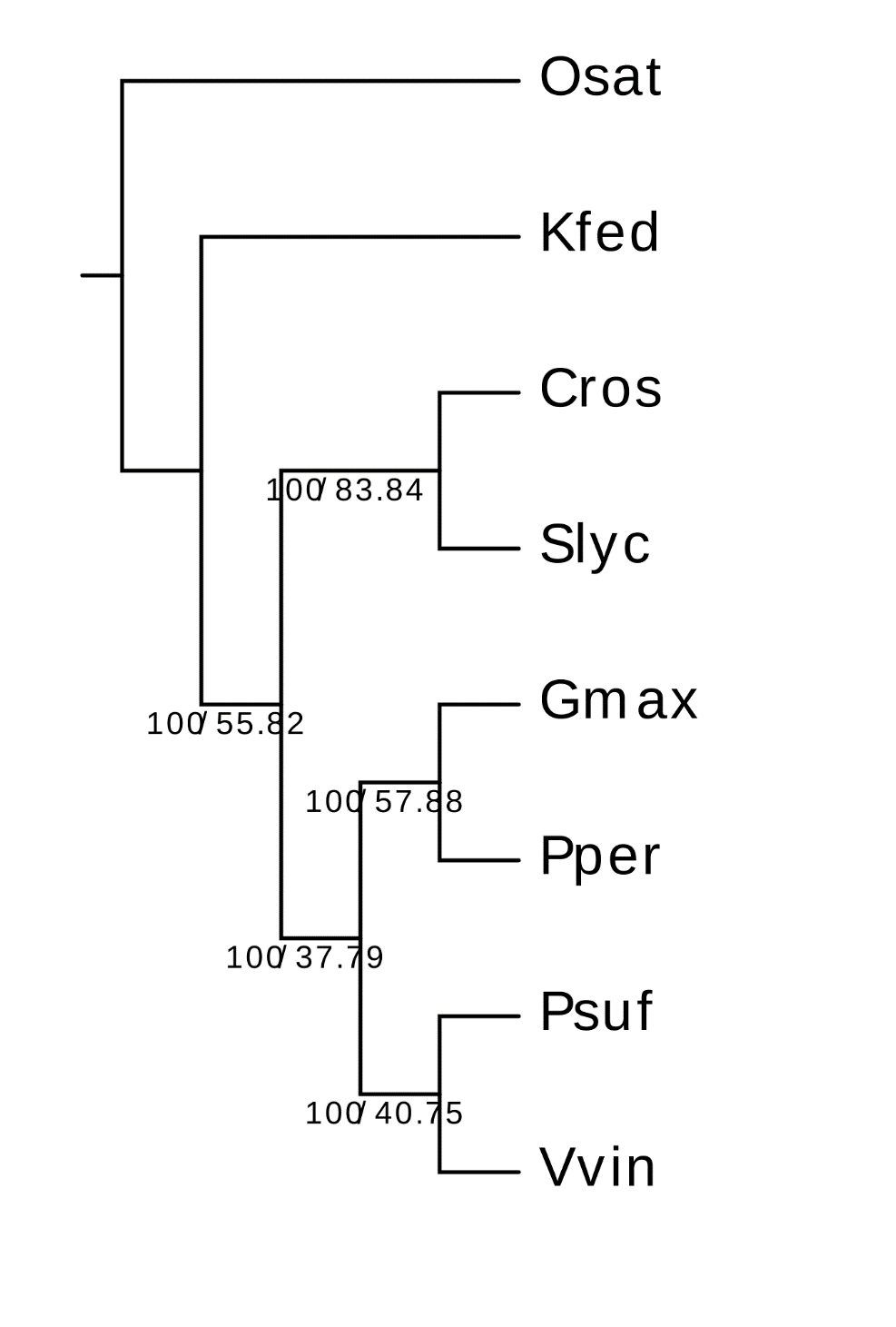


(b)


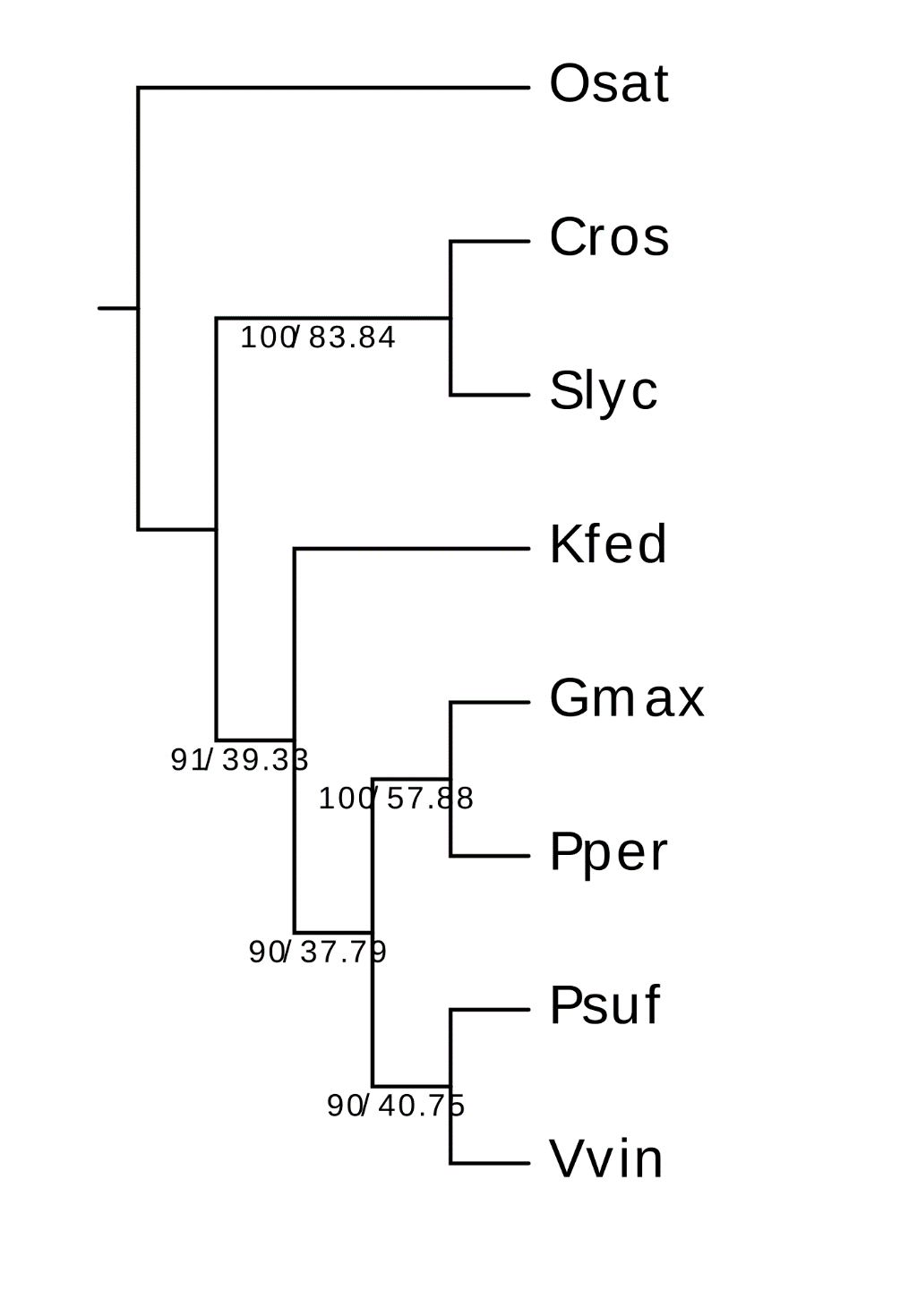


(a)


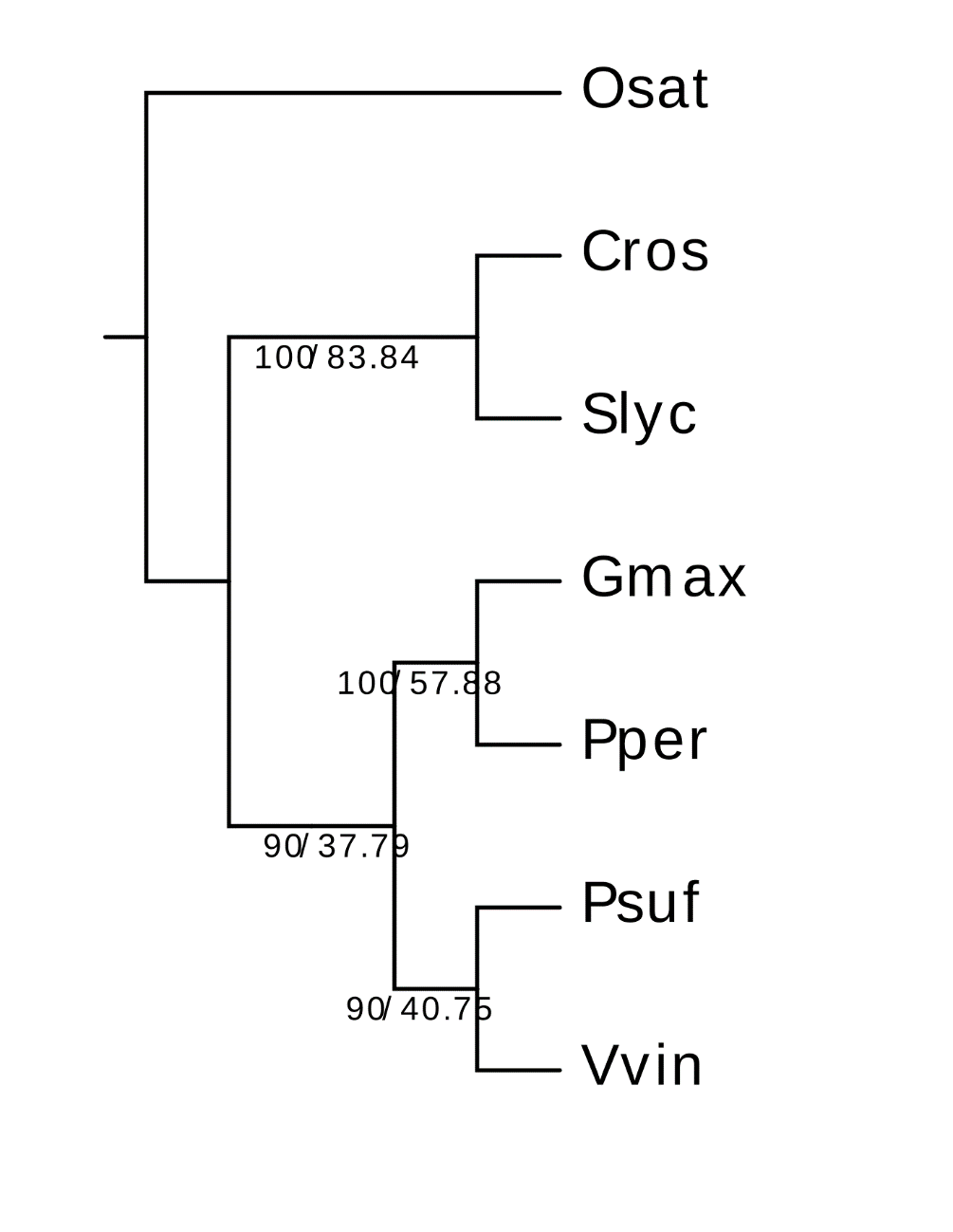

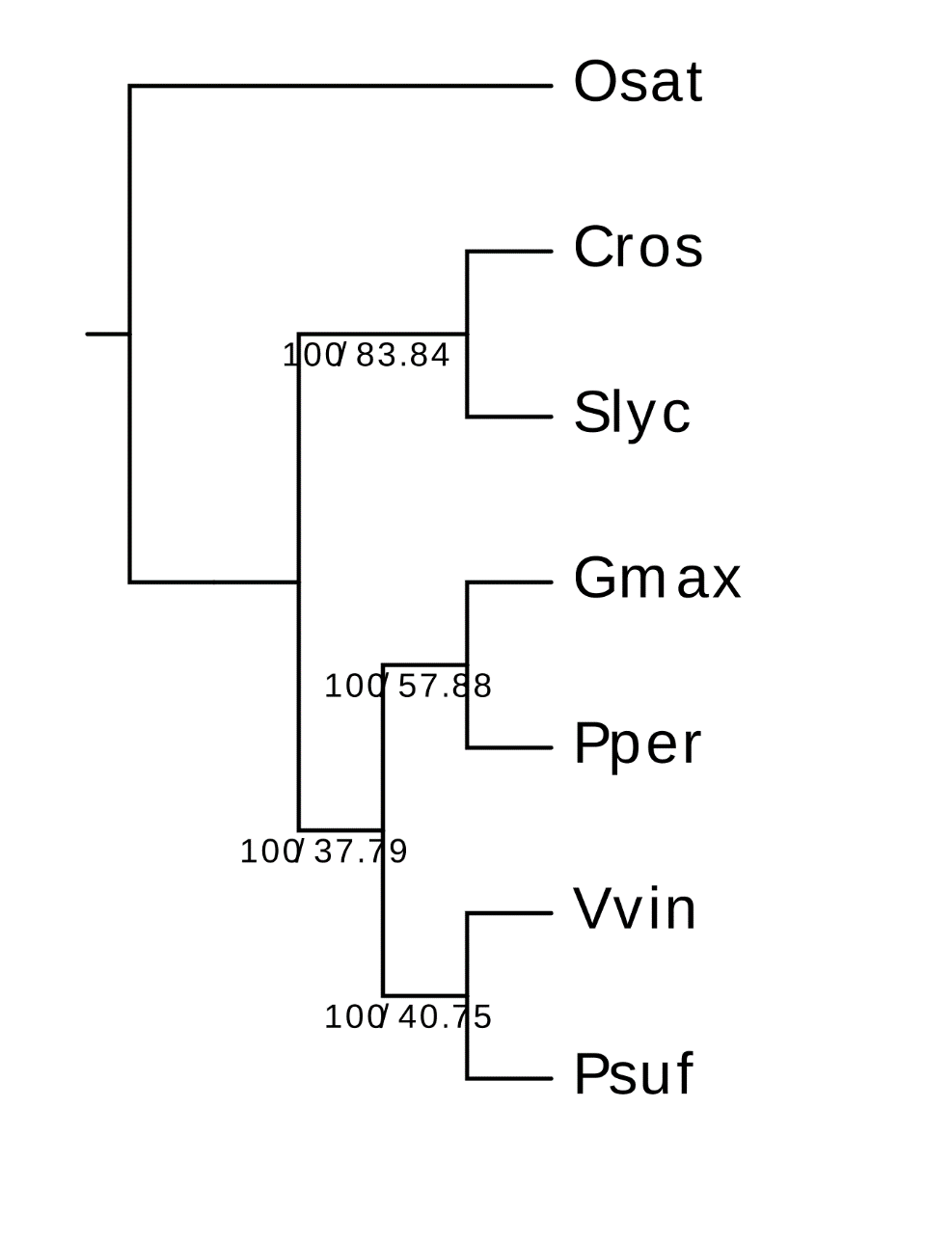


(c)

(d)

**Supplementary Fig. 4 phylogenetic trees reconstructed from 511 single copy genes based on different methods and taxon sets. (a)** **A coalescence-based species tree reconstructed by ASTRAL. (b) A phylogenetic tree created from concatenated protein sequence using maximum-likelihood method. (c) A species tree created with same method used in (a), but *Kalanchoë fedtschenkoi* was not included.** **(d) A phylogenetic tree created with same method used in (b), but *Kalanchoë fedtschenkoi* was not included. Numbers on the left of the nodes are bootstrap support values. Numbers on the right are the proportion of gene trees that support the tree topology. Cros:** ***Catharanthus roseus*, Slyc: *Solanum lycopersicum*, Pper: *Pyunus persica*, Gmax: *Glycine max*, Psuf: *Paeonia suffruticosa*, Vvin: *Vitis vinifera*, Kfed: *Kalanchoë fedtschenkoi*.**


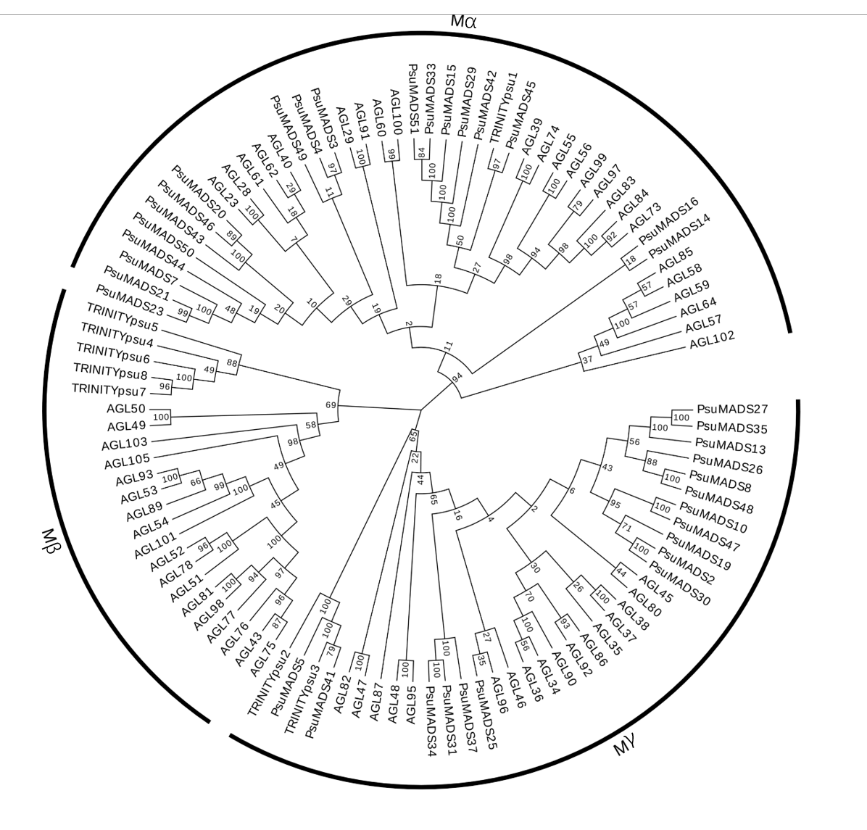


**Supplementary Fig. 5 Phylogenetic tree of *P. suffruticosa* and *Arabidopsis* type I MADS-box genes. The tree was constructed using the neighbor-joining method with 1000 bootstrap replicates. Numbers associated with nodes are bootstrap support values.**


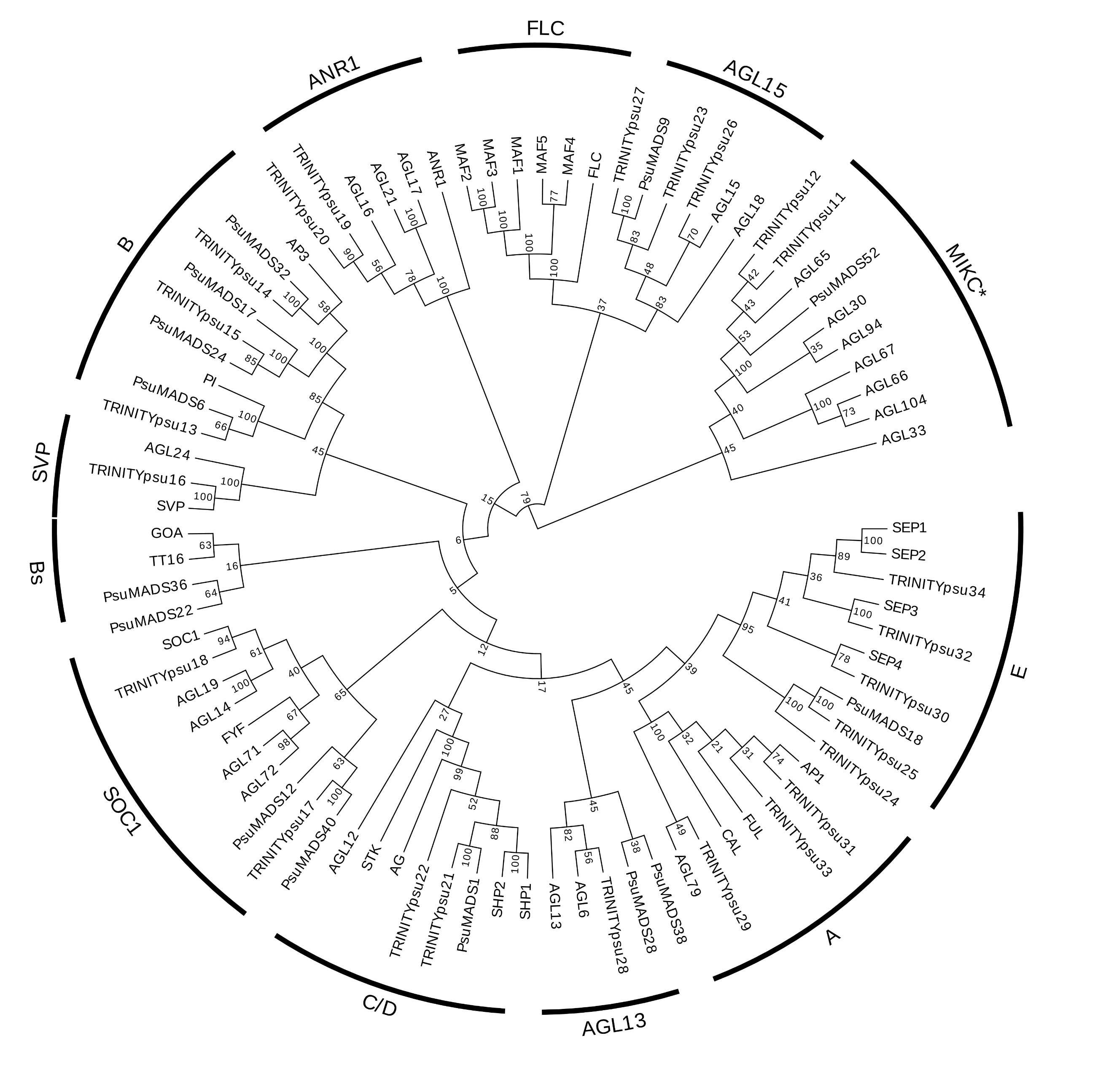


**Supplementary Fig. 6** **Phylogenetic tree of type II MADS-box genes in *P. suffruticosa* and *Arabidopsis*. The tree was constructed using the neighbor-joining method with 1000 bootstrap replicates. Numbers associated with nodes are bootstrap support values.**
